# Cancer-associated fibroblasts reprogram cysteine metabolism to increase tumor resistance to ferroptosis in pancreatic cancer

**DOI:** 10.7150/thno.89805

**Published:** 2024-02-11

**Authors:** Yuchao Zhu, Shuai Fang, Bolin Fan, Kaiwei Xu, Liu Xu, Linwei Wang, Lubin Zhu, Chunqu Chen, Ruoyu Wu, Jiajing Ni, Jianhua Wang

**Affiliations:** 1 Department of Radiology, The First Affiliated Hospital of Xiamen University, 55 Zhenhai Road, Siming District, Xiamen, Fujian Province, People's Republic of China.; 2 Ningbo University Affiliated First Hospital, Ningbo, Zhejiang Province, People's Republic of China.; 3 Ningbo University School of Medicine, Ningbo, Zhejiang Province, People's Republic of China.; 4 State Key Laboratory of Agricultural Microbiology, College of Veterinary Medicine, Huazhong Agricultural University, Wuhan, Hubei Province, People's Republic of China.

**Keywords:** CAF, cysteine, pancreatic cancer, metabolism, TME

## Abstract

**Background:** Pancreatic ductal adenocarcinoma (PDAC) is an insidious, rapidly progressing malignancy of the gastrointestinal tract. Due to its dense fibrous stroma and complex tumor microenvironment, neither of which is sensitive to radiotherapy, pancreatic adenocarcinoma is one of the malignancies with the poorest prognosis. Therefore, detailed elucidation of the inhibitory microenvironment of PDAC is essential for the development of novel therapeutic strategies.

**Methods:** We analyzed the association between cancer-associated fibroblasts (CAFs) and resistance to ferroptosis in PDAC using conditioned CAF medium and co-culture of pancreatic cancer cells. Abnormal cysteine metabolism was observed in CAFs using non-targeted metabolomics analysis with liquid chromatography-tandem mass spectrometry (LC-MS/MS). The regulatory effects of cysteine were investigated in PDAC cells through measurement of cell cloning, cell death, cell function, and EdU assays. The effects of exogenous cysteine intake were examined in a mouse xenograft model and the effects of the cysteine pathway on ferroptosis in PDAC were investigated by western blotting, measurement of glutathione and reactive oxygen species levels, among others.

**Results:** It was found that CAFs played a critical role in PDAC metabolism by secreting cysteine, which could increase tumor resistance to ferroptosis. A previously unrecognized function of the sulfur transfer pathway in CAFs was identified, which increased the extracellular supply of cysteine to support glutathione synthesis and thus inducing ferroptosis resistance. Cysteine secretion by CAFs was found to be mediated by the TGF-β/SMAD3/ATF4 signaling axis.

**Conclusion:** Taken together, the findings demonstrate a novel metabolic relationship between CAFs and cancer cells, in which cysteine generated by CAFs acts as a substrate in the prevention of oxidative damage in PDAC and thus suggests new therapeutic targets for PDAC.

## Background

Pancreatic ductal adenocarcinoma (PDAC) is a leading cause of cancer-related mortality worldwide, with limited therapeutic options and poor long-term survival^1^. Approximately 80% of patients with PDAC have locally advanced or metastatic disease at presentation and are not candidates for curative intent surgery, making systemic therapy the mainstay of treatment^2^. The poor prognosis of patients with PDAC is associated with the highly fibrous matrix of the tumor which is resistant to radiotherapy and chemotherapy^3^. PDAC has a complex tumor microenvironment (TME), which consists of extracellular matrix (ECM) proteins, fibroblasts, endothelial cells, pericytes, neurons, and immune cells that have infiltrated the tissue^2^, ^4^. The dense matrix contains the tumor epithelium, and fibroblasts are thought to be the primary mediators of disease progression through direct interaction with cancer cells. The neoplastic epithelium exists within a dense stroma composed mainly of fibroblasts, which are recognized as critical mediators of disease progression through their direct effects on cancer cells^5^, ^6^.

Reprogramming of cellular metabolism is necessary for the initiation and growth of malignant tumors. Cancer cells engage in metabolic reprogramming to obtain sufficient energy or extra nutrients, especially in the presence of nutrient deprivation, to maintain rapid cell growth and reduce cell pressure^7^. To meet their metabolic needs, malignant tumors must therefore alter the ways in which they acquire and utilize nutrients. These metabolic changes allow the tumor cells to utilize both conventional and unconventional sources of nutrition, using these resources to support the growth of both tumor cells and the TME^2^. Both the development and subsequent growth of tumors are supported by cancer-associated fibroblasts (CAFs) in various ways. CAFs modify the ECM through the secretion of remodeling enzymes as well as collagen and laminin. CAFs can also promote tumor angiogenesis through the production of a range of pro-inflammatory interleukins, tumor necrosis factor (TNF), transforming growth factor (TGF), fibroblast growth factor (FGF), and vascular endothelial growth factor VEGF)^8^, ^9^. Increasing evidence suggests that metabolites released by CAFs are also important sources of nutrients that support numerous metabolic processes in cancer and provide the metabolic requirements for the rapid growth of tumor cells^10^.

The non-essential amino acid cysteine is important for mammalian cells as its thiol group is the basis for several cellular antioxidant defense mechanisms, including the glutathione (GSH) system. Cysteine is associated with a number of oncoproteins, tumor suppressors, and malignant features of cancer^11^. The development of malignancy requires that tumor cells overcome the barriers of oxidative stress at various stages of tumorigenesis and, therefore, the maintenance of an adequate supply of cysteine is essential to regulate the balance between oxidative damage and antioxidant defenses in cancer progression^12^. As cysteine is often a limiting nutrient in the TME, ensuring its supply to sustain cell growth in the face of cysteine limitation is critical^13^. Therefore, stromal cysteine production and secretion via transsulfuration may be required to support tumor growth. A recent study has shown that PDAC cells can regulate the cysteine transporter SLC7A11, which controls cysteine uptake, by using autophagy mechanisms^14^.

**Figure 1:**
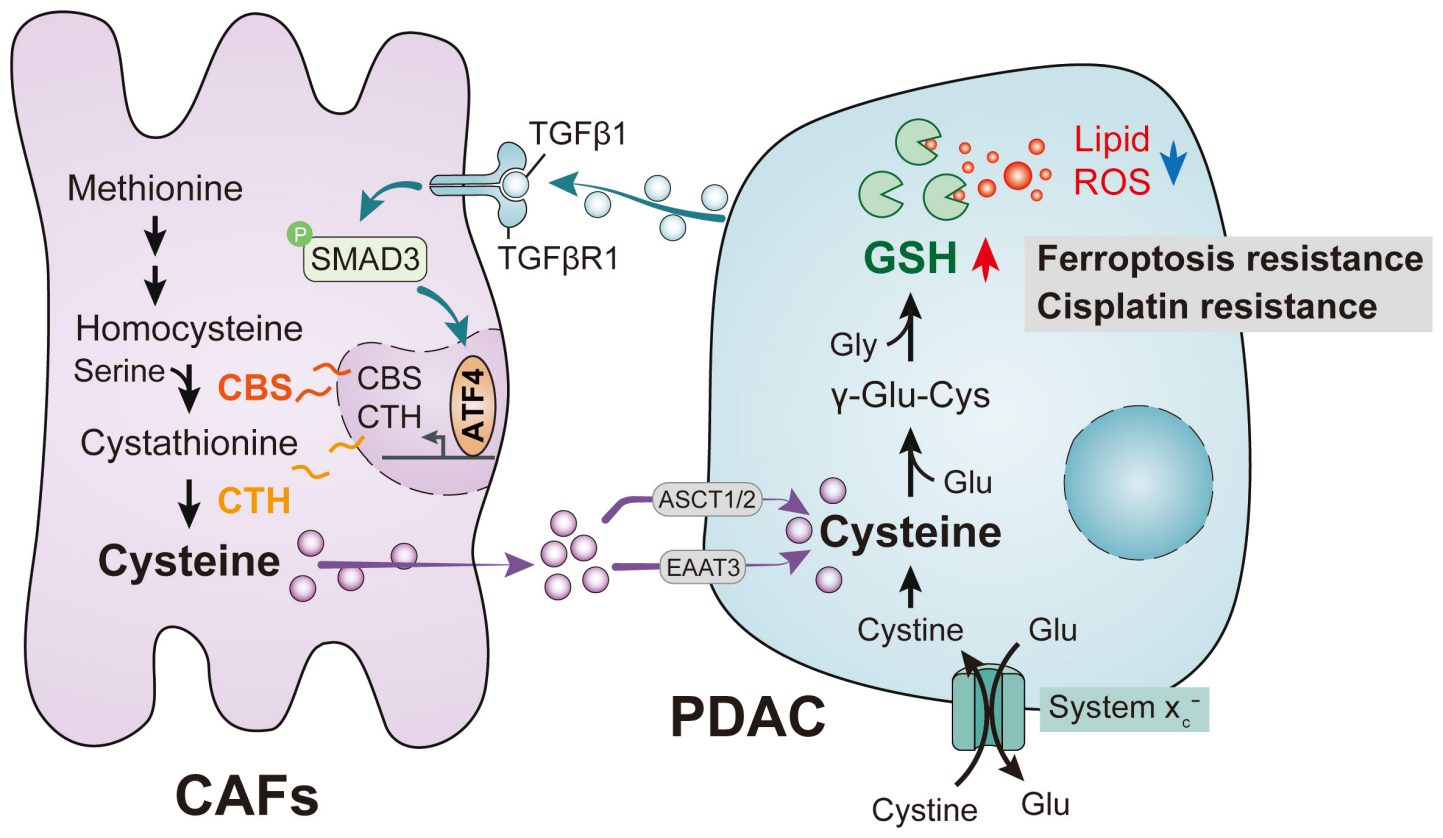
PDAC mediates abnormal CAF cysteine metabolism via the TGF-β/SMAD3/ATF4 signaling axis, thereby inducing ferroptosis resistance.

A recently discovered form of programmed cell death, ferroptosis, is primarily dependent on iron-mediated oxidative damage and subsequent disruption of the cell membrane^15^. Ferroptosis is triggered by iron-dependent phospholipid peroxidation and is controlled by a variety of cellular metabolic processes, including redox homeostasis, iron handling, mitochondrial activity, amino acid, lipid and sugar metabolism, and numerous disease-related signaling pathways^16^. Many drug-resistant cancer cells have been, surprisingly, found to be highly susceptible to ferroptosis, particularly cells that are mesenchymal and prone to metastasis^15^, ^17^, ^18^. Therefore, altering the balance between oxidative damage and antioxidant defense mechanisms to induce ferroptosis is a potential therapeutic opportunity to reduce cancer development. Interestingly, CAFs have been shown to interfere with the redox balance to influence ferroptosis in tumor cells. Recent studies have shown that CAFs secrete the microRNA miR-522 in extracellular vesicles to inhibit iron-dependent death in cancer cells by targeting ALOX15 and blocking the accumulation of lipid-associated reactive oxygen species (ROS)^19^; CAFs also enhance HSF1 activation and increase DLEU1 transcript levels to regulate Erastin-induced ferroptosis in tumor cells^20^ and can promote chemical resistance in PDAC cells after gemcitabine treatment by signaling involving extracellular vesicle-derived ACSL4^21^. In the present study, we demonstrated that CAFs play a critical role in PDAC metabolism by secreting cysteine, which increases tumor resistance to ferroptosis. Specifically, we identified a previously unrecognized function of CAFs and the CAF transsulfuration pathway, which increases extracellular cysteine supply in PDAC to support the synthesis of glutathione (GSH) and thereby prevent PDAC ferroptosis. Additionally, we showed that activation of TGF-/SMAD3/ATF4 signaling is required for cysteine secretion by CAFs. Our findings thus revealed a novel metabolic relationship between CAFs and cancer cells, where CAF-generated cysteine serves as a substrate to prevent oxidative damage in PDAC (Figure 1).

## Methods

### Human tissue samples and ethics statement

Fifty-six tissue samples, comprising both tumor and normal adjacent tissue, were procured from pancreatic cancer patients at Ningbo Clinical Pathological Diagnosis Center and Ningbo University Hospital. The samples were obtained through surgical resection and were sourced from patients who had not undergone radiotherapy, chemotherapy, or targeted therapy prior to the surgery. Clinical information and case data were collected from the patients before the surgical procedure. All patients provided written informed consent, and the experimental protocol was approved by the Medical Ethics Committee of Ningbo University School of Medicine.

### Cell culture

MIAPaCa-2, PANC-1, AsPc-1, and PaTu8988t cells were purchased from the Cell Bank of the Chinese Academy of Sciences (Shanghai, China) and tested negative for mycoplasma. Cells were grown in an incubator at 5% CO_2_ and 37 ℃. The cell lines were cultured in DMEM (Gibco, USA) containing L-glutamine, 4.5 g/L D-glucose, and 110 mg/L pyruvate nano, together with 1% penicillin-streptomycin (HYClone, USA) and 10% fetal bovine serum (FBS, Gibco). For cell starvation, the cells were washed twice with PBS before culture in serum-free DMEM.

### Isolation and culture of fibroblasts

CAFs were isolated from pancreatic cancer tissue, while normal fibroblasts (NFs) were obtained from normal tissue at least 2 cm away from pancreatic cancer tissue. The tumor and paracancerous tissues were ground into small pieces using a tumor isolation kit (Miltenyi, Germany) and digested to produce single-cell suspensions. The fibroblasts were then isolated using the differences in adhesion between epithelial cells and fibroblasts and the difference in the separation time. After trypsin digestion CAFs and NFs were raised in DMEM/F12 medium and cultured for 2-3 generations.

### RNA extraction and RT-qPCR

Total RNA was extracted from cancer tissues and cell lines using TRIzol reagent (Invitrogen, USA) and was reverse-transcribed using a reverse-transcription kit (Toyobo, Japan). RT-qPCR was performed using SYBR Green (Takara, Japan). β-actin was used as an internal standard control. The specific primers used are listed in Table S1 (Additional file 1).

### Cell viability assay

Cell viability was assayed using Cell Counting Kit 8 (CCK-8) (Yeasen, China) according to the manufacturer's guidelines. MIAPaCa-2 and PANC-1 cells were inoculated into 96-well plates at densities of 5000 cells/well, after which 10 μL of the CCK-8 reagent was added to each well, gently mixed, and incubated for 4 h under standard conditions, and the absorbance of each well was measured at 450 nm.

### Cell cloning

MIAPaCa-2 and PANC-1 cells were inoculated into six-well plates at 1500 cells per well. Two milliliters of medium were added to each well, and the culture was incubated for 12 days under standard conditions. The experiment was halted when obvious cell clusters were seen. The cells were fixed with 4% paraformaldehyde (Solarbio, China), stained with crystal violet (Solarbio), and counted.

### Cell death assays

Cells were digested with trypsin and washed with PBS, before dual-staining with Annexin V and PI using Annexin V/FITC. The cells were then evaluated on a Beckman CytoFlex S flow cytometer (USA) within half an hour of staining. The data were analyzed using FlowJo v10.

### LC-MS/MS analysis

The culture supernatants of isolated CAFs and NFs were collected (6 mL) and clarified by centrifugation. The LC-MS/MS was performed by OE Biotech (Shanghai, China) using a liquid-liquid mass spectrometry system consisting of an ACQUITY UPLC I-Class plus liquid-phase tandem AB ExionLC mass spectrometer. MS data were analyzed using Progenesis QI software version 2.3.

### Glutathione determination

Cells (1 x 10^5^ cells per well) were inoculated into a 10 cm plate. The cells were washed with ice-cold PBS, harvested, and deproteinized with 5% 5-sulfosalicylic acid solution (SSA)(Sigma-Aldrich). Total glutathione (reduced glutathione [GSH] plus glutathione disulfide [GSSG]) was measured spectrally at 412nm using a glutathione determination kit (CS0260, Sigma-Aldrich), according to the manufacturer's instructions.

### Malondialdehyde (MDA) assay

MDA concentrations were measured using an MDA assay kit (Solarbio). 1*10^5^ cells/well density were inoculated into 10cm plates. The cells were lysed and centrifuged, and the absorbance was measured at 532 nm after the addition of MDA assay working solution at 100℃.

### ROS assay

A Reactive Oxygen Species Assay kit (Solarbio) was used to examine the production of intracellular ROS, following the manufacturer's guidelines. Cells were inoculated into six-well plates and were incubated with a DCFH-DA fluorescent probe solution for 20 min. The fluorescence of the cells was measured using a flow cytometer (Beckman).

### 5-Ethynyl-20-deoxyuridine (EdU) Assay

The Cell-Light EdU DNA Cell Proliferation Kit (Beyotime, China) was used for the EdU assay according to the manufacturer's guidelines. Cells were inoculated into six-well plates. Edu working solution was added to the plates and incubated for 2 h, after which the cells were fixed with 4% paraformaldehyde for 15 min. The Click working solution was added for light-protected incubation, with the further addition of Hoechst 33342 after 30 min. The cells were incubated for 10 min at room temperature with protection from light, and were then evaluated and imaged using a fluorescent microscope (Olympus, Japan).

### Xenograft mouse tumor model

The animal experiments were approved by the Animal Research Ethics Committee of the Affiliated Hospital of Ningbo University School of Medicine. BALB/c-Nude mice were purchased from Beijing Viton Lever and randomly grouped. PANC-1 or MIAPaCa-2 cells (1× 10^7^ cells per mouse) transfected with shCBS vector or empty vector were injected into the right posterior abdomen of 6-week-old nude mice. Half of the mice received cysteine supplementation in their diet. Tumor sizes were recorded each week and the tumor volumes were calculated as length×width^2^×0.5. After five weeks, the mice were euthanized and the tumors were collected for further testing.

### Western blotting

The total protein contents of the cells or tissues were obtained after lysis in RIPA buffer (Solarbio) containing protease inhibitors. The proteins were then boiled in SDS sample buffer and separated on SDS-PAGE before transfer to PVDF membranes (Millipore, USA). After blocking with 5% skim milk, the membranes were treated with primary antibodies at 4℃ overnight. Antibodies against the following proteins were used: SLC7A11 (Proteintech, China), CBS (Proteintech), CTH (Abcam, UK), ATF4 (Abcam), γH2AX (Abcam), p-SMAD3 (Proteintech), SMAD3 (Proteintech), GAPDH (Proteintech), and β-actin (Abcam). The membranes were then incubated with secondary antibodies for 2 h. The bands were visualized using the ECL detection reagent (Advansta, USA).

### Immunohistochemistry

Appropriately sized tumor samples were fixed in 4% paraformaldehyde, paraffin-embedded, sectioned, and incubated with primary antibodies against Ki67 (Abcam), γH2AX (Abcam), CBS (Proteintech) overnight at 4 ℃. Incubation with secondary antibodies was performed at room temperature. Staining was performed using DHB solution, and hematoxylin solution was used to stain the nuclei. The slides were examined and imaged under microscopy (Leica, Germany).

### Immunofluorescence

Cell crawls were seeded in well plates for cell culture. The cells were fixed in 4% paraformaldehyde for 15 min, followed by blocking with 3% BSA. The cells were then incubated with the anti-SMAD3 antibody at 4 ℃. The nuclei were counterstained with 4′,6-diamidino-2-phenylindole (DAPI) for 1 h in the dark, and the cells were examined and imaged under confocal microscopy (Leica).

### CUT&Tag

Cells were collected and the procedure involved the permeabilization of cells and subsequent immobilization on concanavalin A-coated magnetic beads to aid in the washing steps. Cells were then incubated with a negative control IgG or a primary antibody protein, followed by incubation with a secondary antibody. This was followed by the incubation with assembled transposomes, which consisted of protein A fused to the Tn5 transposase enzyme conjugated to NGS adapters. After stringent washing to remove unbound transposomes, the reaction was activated by the addition of Mg_2_. This led to chromatin cleavage near the protein binding site and simultaneous addition of NGS adapter DNA sequences, facilitating chromatin cleavage and library preparation in a single step. All steps were performed according to the manufacturer's instructions.

### Statistical analysis

Data were analyzed using GraphPad Prism 8 software and are expressed as mean ± standard deviation. Differences were considered significant at *P* < 0.05 with * indicating* p* < 0.05, ** indicating *p* < 0.01, *** indicating* p* < 0.001.

## Results

### CAFs are associated with ferroptosis resistance in PDAC

**Figure 2:**
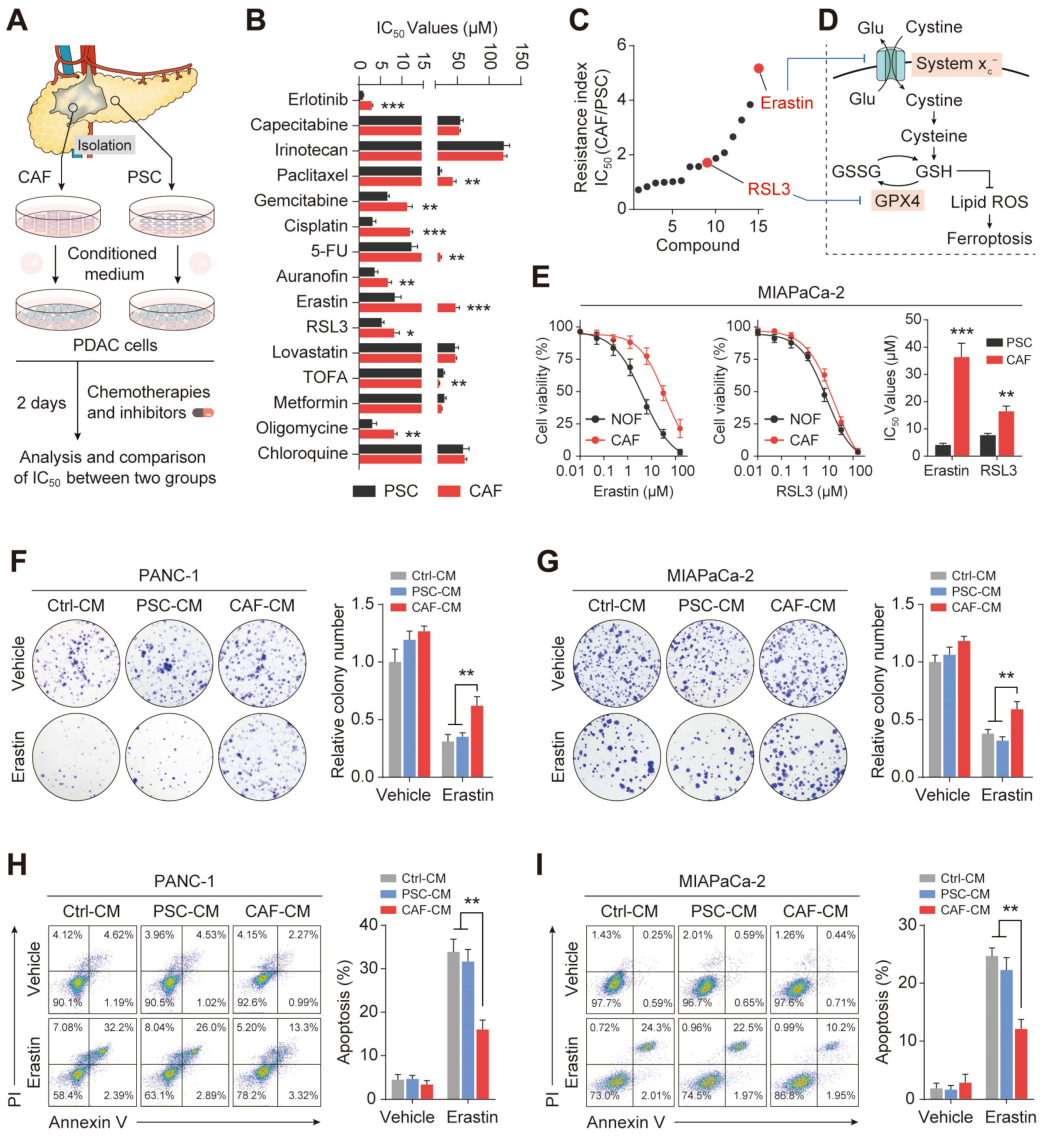
** CAFs are associated with ferroptosis resistance in PDAC. Culture of PDAC cells with CAFS-CM or PSCs-CM.** (A) Flow chart to verify drug sensitivity in PANC-1 cells. (B-C) CCK-8 assays were used to measure cell viability after treatment with metabolic inhibitors or chemotherapy drugs. (D) Schematic diagram of ferroptosis-associated metabolic pathways. (E) Viability in MIAPaCa-2 cells was measured by CCK-8 assays after Erastin or RSL3 treatment. (F-G) Colony formation in PANC-1 and MIAPaCa-2 cells was evaluated by clonogenesis assays after Erastin or vehicle treatment. (H-I) Cell death ratios in PANC-1 and MIAPaCa-2 cells were evaluated by cell cell death assays after Erastin or vehicle treatment. * P < 0.05, ** P < 0.01, *** P < 0.001.

To determine the role of stromal CAFs in the metabolic remodeling of PDAC, metabolic vulnerabilities induced by CAFs that could be targeted in antitumor therapeutics were investigated. To test this, we examined the sensitivity of PDAC cells to a panel of metabolism-targeted small molecules. PANC-1 cells were treated with conditioned medium (CM) from CAFs or normal human pancreatic stellate cells (PSCs), derived from patients with PDAC, followed by two days of drug treatment (Figure 2A). Consistent with previous evidence, it was found that the presence of CAFs protected PDAC cells during treatment with metabolic inhibitors and chemotherapeutic drugs, demonstrated by increased IC_50_ values (Figure 2B). Notably, we observed that the top desensitizing agent was Erastin, an inhibitor of the system xc^-^ cystine/glutamate antiporter. The results also showed that CAFs protected PDAC cells after treatment with the ferroptosis inducer RSL3 (Figure 2C-D and Figure S1A). Although erastin and RSL3 are both ferroptosis inducers, inconsistently, the IC50 effect of CAF on erastin is much more significant than that of RSL3. This may be due to the fact that Erastin did not affect the uptake of CAF- derived cysteine by PDACs orCAF - derived cysteine is eventually synthesized into glutathione in PDACs, which could significantly be inhibited by RSL3.

To further confirm the potential role of CAFs in PDAC ferroptosis, another PDAC cell line, MIAPaCa-2, was cultured with conditioned medium from CAFs or PSCs followed by Erastin and RSL3 treatment. As expected, the CAF-CM treatment significantly increased the IC_50_ values of these two ferroptosis inducers, especially Erastin (Figure 2E). This CAF-induced Erastin insensitivity was confirmed using imidazole ketone Erastin (IKE), an Erastin analog (Figure S1B). Functionally, after treatment with CAF-CM, colony formation was promoted in PDAC cells, with more colonies surviving after the Erastin exposure (Figure 2F-G). Flow cytometry-based Annexin-V/propidium iodide (PI) cell death assays demonstrated that CAF-CM treatment dramatically reduced Erastin-induced cell death in both PANC-1 and MIAPaCa-2 cells (Figure 2H-I). Consistently, compared with PSC-CM, PDAC cells incubated with CAF-CM formed greater numbers of colonies while showing reduced drug-induced cell death rates after Erastin or RSL3 treatment (Figure S1C-D). These findings suggest that CAFs play a critical role in ferroptosis in PDAC.

### Exocrine cysteine is essential for CAF-induced ferroptosis resistance in PDAC

**Figure 3:**
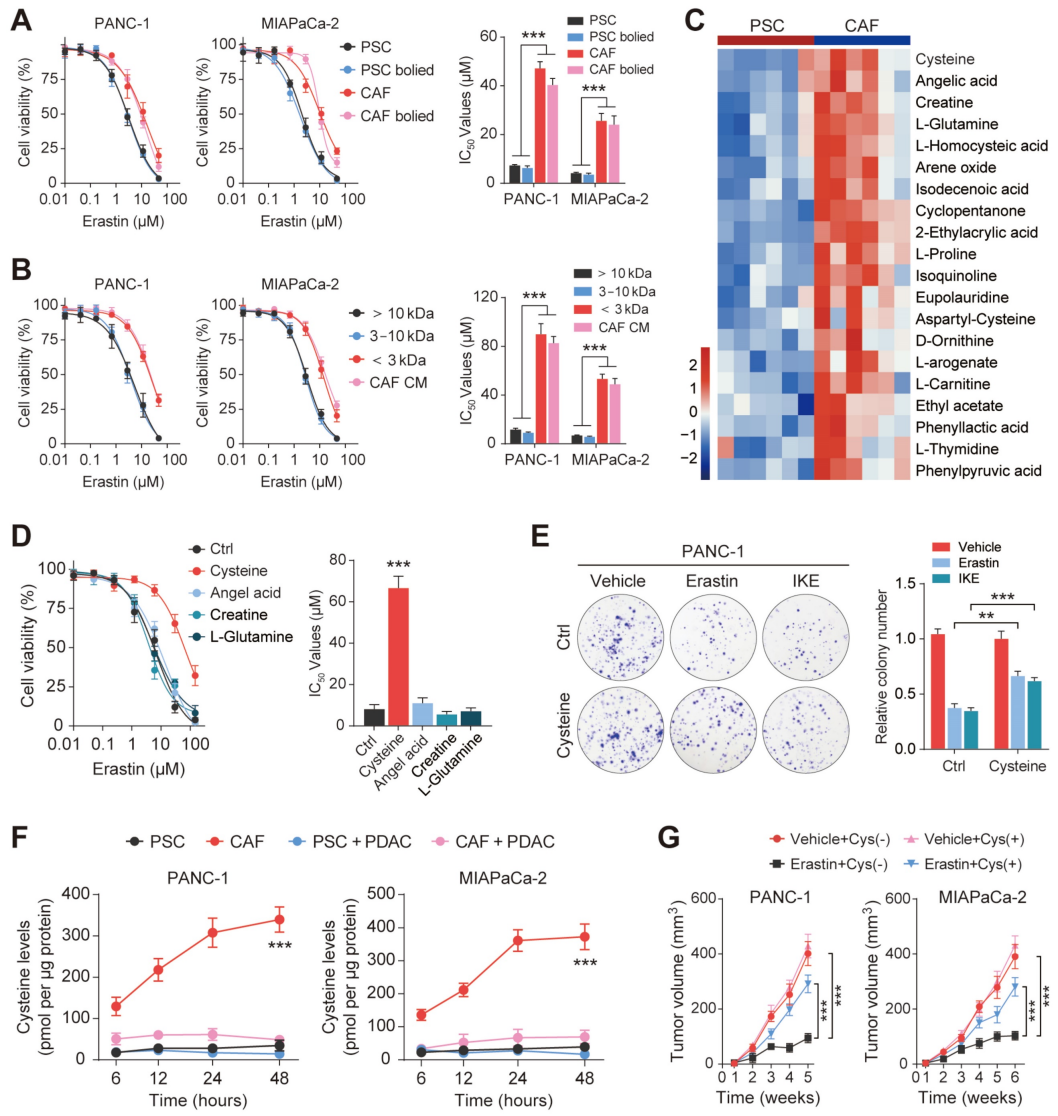
** Exocrine cysteine is essential for CAF-induced ferroptosis resistance in PDAC.** (A) Cell viability was measured by CCK-8 assays in PANC-1 and MIAPaCa-2 cells lines cultured in CAF-CM, CAF-CM, PSC-CM, or PSC-CM after boiling at 100 ℃. (B) Cell viability was measured by CCK-8 assays in PANC-1 and MIAPaCa-2 cells lines cultured with < 3 kDa, 3-10 kDa, >10 kDa, or untreated CAF-CM and treated with Erastin. (C) Cluster heatmap of CAF-CM and PSC-CM metabolites. (D) Cell viability in PCK-8 cells cultured with cysteine, angelic acid, creatine, or glutamine, and treated with Erastin. (E) Colony formation in PANC-1 cells cultured with Ctrl or cysteine and treated with Erastin or IKE. (F) Cysteine levels in PANC-1 and MIAPaCa-2 cells after 6, 12, 24 and 48 h. (G) Tumor volumes in xenograft mice (N=6). ** P < 0.01, *** P < 0.001.

Previous studies have evaluated the function of CAFs in promoting antitumor therapeutic resistance by generating soluble factors that can act on cancer cells through paracrine mechanisms^19^, ^22^. Therefore, we hypothesized that CAFs regulate PDAC ferroptosis resistance by secreting metabolism-associated factors. To verify this hypothesis, we first subjected the conditioned medium to heating (100 ℃, 15 min) or three freeze-thaw cycles (-80 ℃, 60 ℃) and observed that it retained the ability to promote ferroptosis resistance in PDAC, indicating that the factor(s) lacked tertiary structure (Figure 3A and Figure S2A). Moreover, we collected and fractioned CAF CM into fractions of <3 kDa, 3-10 kDa, and >10 kDa using specific filters and centrifugation. Only the <3 kDa fraction of the CAF-CM retained the ability to resist Erastin in PANC-1 and MIAPaCa-2 cells (Figure 3B). Therefore, the small size and resistance to extreme temperatures of the target factors excluded large candidate molecules such as proteins, indicating that the factors could be a metabolite or metabolites.

Untargeted metabolomic analysis using liquid chromatography coupled to tandem mass spectrometry (LC-MS/MS) was performed to detect metabolic profile changes in the CAF-CM and PSC-CM. This identified cysteine, angelic acid, creatine, and glutamine as the CAF metabolites showing the most significant increases compared with PCSs (Figure 3C). Interestingly, a growing body of evidence has shown that certain cancers, including PDAC, import cysteine (cystine) via system x_C_^-^ to synthesize glutathione and coenzyme A, which, together, down-regulate ferroptosis^23^. To investigate the potential roles of cysteine, angelic acid, creatine, and glutamine in promoting ferroptosis resistance, these small molecules were included in the medium of PANC-1 cells treated with Erastin. It was observed that only cysteine enhanced the proliferation rate and significantly increased the IC_50_ values of Erastin in PANC-1 cells (Figure S2B, Figure 3D). In addition, colony formation assays and flow cytometry further confirmed that cysteine supplementation enhanced ferroptosis resistance in PDAC cells treated with Erastin or IKE (Figure 3E, Figure S2C-E).

Since the results of metabolomic analyses do not always accurately translate to the metabolic phenotype, we measured the exocrine levels of cysteine in CAFs and PSCs. We observed that CAFs secreted significantly greater amounts of cysteine than PSCs, which could then be efficiently taken up by PDAC cells (Figure 3F). PDAC cells were then inoculated subcutaneously into BALB/c-Nude mice for the in vivo study. The results showed that vehicle PDAC cell-derived xenografts (cysteine-free diet) responded well to Erastin, as shown by decreased tumor volumes after drug treatment. In contrast, the tumors of the animals fed a cysteine-supplemented diet were much less sensitive to Erastin (Figure 3G). Moreover, Ki-67 staining showed that Erastin inhibited PDAC cell proliferation less effectively in the mice receiving supplementary cysteine compared with the cysteine-free group (Figure S2F). Collectively, these data suggest that cysteine, produced predominantly by CAFs, contributes to CAF-induced ferroptosis resistance in PDAC.

### Enhanced transsulfuration activity contributes to de novo cysteine synthesis in CAFs

**Figure 4:**
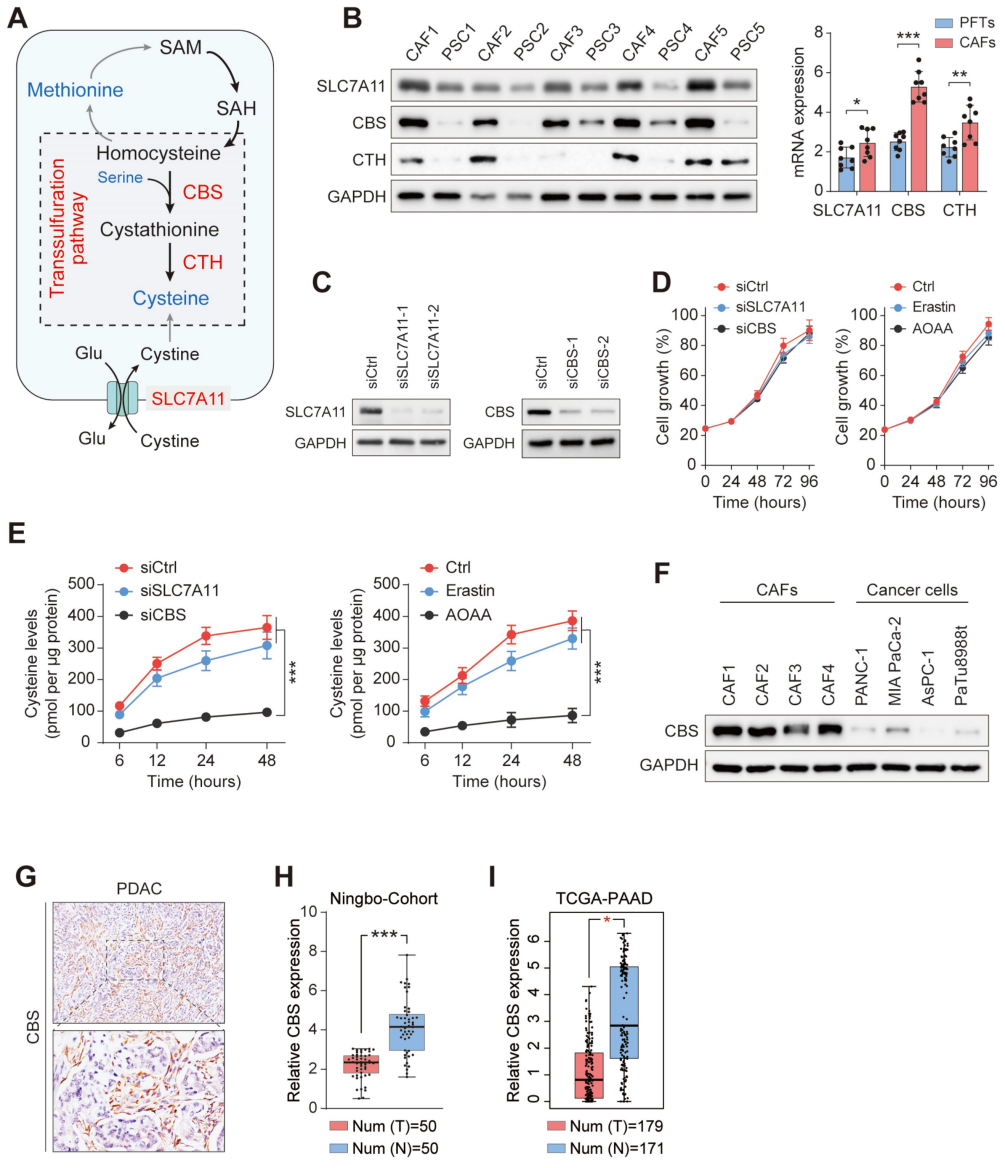
** Enhanced transsulfuration activity contributes to de novo cysteine synthesis in CAFs.** (A) Schematic diagram of cysteine metabolism in cells. (B) qRT-PCR or Western blotting assays of CBS, CTH, and SLC7A11 expression in five pairs of CAFs and matched PSCs. (C) SLC7A11 and CBS expression after siRNA treatment shown by western blotting. (D) Cysteine levels in PANC-1 and MIAPaCa-2 cells after treatment with Ctrl, Erastin, or AOAA. (E) Viability of cells after siRNA or inhibitor treatment, shown by CCK-8 assays. (F) CBS expression in CAFs and PDAC cell lines, shown by western blotting. (G) CBS expression in tissues, shown by immunohistochemistry. (H) CBS expression in pancreatic cancer tissues and paracancerous tissues, shown by qRT-PCR. (I) CBS expression in 179 pancreatic cancer tissues and 171 normal tissues from the TCGA database. * P < 0.05, ** P < 0.01, *** P < 0.001.

Cysteine is an amino acid required by proliferating cells both as a building block for protein production and as a substrate to avoid oxidative damage^11^. To investigate the sources of exocrine cysteine in CAFs, we first examined the expression levels of CBS and CTH, two enzymes in the transsulfuration pathway, and the expression level of SLC7A11, the regulatory component of the system xc- amino acid antiporter (Figure 4A). CAFs and matched PSCs from five patients with PDAC showed, compared with PSCs, significantly enhanced expression of CBS and CTH and slightly increased levels of SLC7A11 (Figure 4B). This result suggested that the CAF-secreted cysteine is mainly synthesized endogenously via the transsulfuration pathway. To test this prediction, we disrupted SLC7A11 and CBS with small interfering RNA in CAFs (Figure 4C), and measured the exocrine levels of cysteine in CAFs treated with SLC7A11 inhibition (siSLC7A11 or Erastin) or CBS inhibition (siCBS or aminooxyacetic acid, AOAA, a CBS inhibitor). Results showed a significant decline in the exocrine cysteine level only in CAFs treated with CBS inhibition, while disruption of either SLC7A11 or CBS in CAFs had no significant effect on cell proliferation (Figure 4D-E).

The protein expression of CBS was then assessed in CAFs and PDAC cells to determine differences in cysteine anabolism. Notably, it was found that CAFs had substantially higher CBS expression than PDAC cell lines (Figure 4F). Immunohistochemical staining further verified that the stromal component expressed substantially more CBS than its epithelial counterpart in human PDAC (Figure 4G). In addition, RT-PCR analysis showed that CBS expression was significantly reduced in PDAC tissues compared with their levels in normal pancreatic tissues (Figure 4H). The higher expression of CBS in PDAC was then confirmed in data from The Cancer Genome Atlas (TCGA) (Figure 4I). The finding that cancer cells have reduced CBS anabolism compared to that in CAFs and normal pancreatic cells suggests that the exocrine cysteine produced by CAFs has a major role in maintaining metabolic activity in the cysteine/cystine-starved pancreatic milieu.

### Stromal de novo CBS-dependent cysteine biosynthesis is required for ferroptosis resistance in PDAC

**Figure 5:**
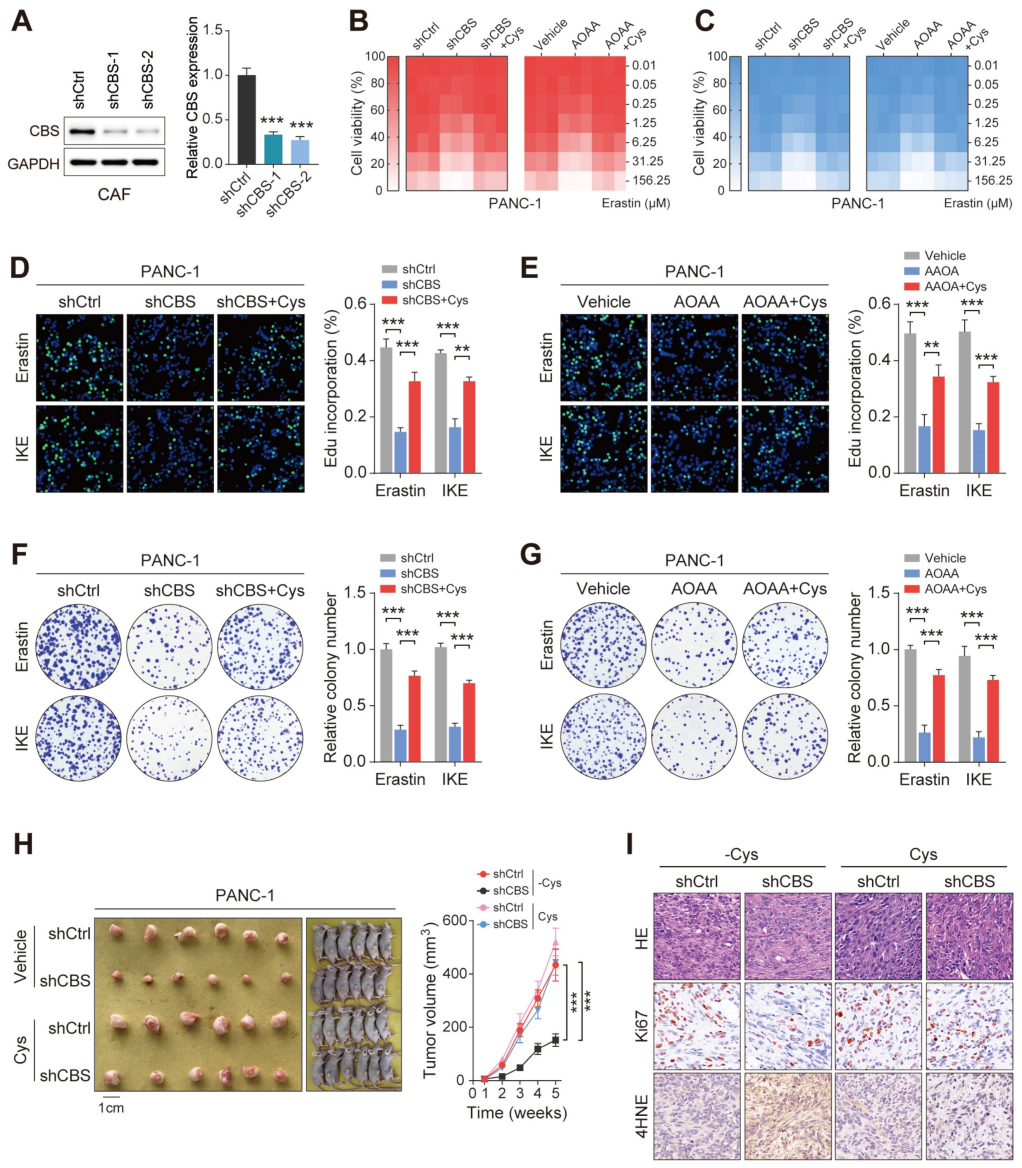
** Stromal CBS-dependent de novo biosynthesis of cysteine is required for ferroptosis resistance in PDAC.** (A) Construction of two CBS-knockdown CAF cell lines using shRNA with measurement of CBS expression by Western blotting and RT-PCR. (B-C) CCK-8 measurement of cell viability. (D-E) EdU assay results of cell proliferation. (F-G) Colony formation by cells. (H) Images and volumes of xenograft tumors (N=6). (I) Representative H&E-stained micrographs of mouse xenograft tumors showing IHC staining of Ki67 and 4HNE. Functional experiments use shCBS-2 for knockdown of CBS. ** P < 0.01, *** P < 0.001.

The finding that enhanced transsulfuration activity contributes to the exocrine secretion of cysteine in CAFs raises the possibility that stromal CBS may participate in the evasion of PDAC ferroptosis through regulation of the metabolic state of the tumor. To further confirm the potential role of stromal CBS in PDAC ferroptosis, we generated stably CBS-depleted CAF cells using two independent shRNAs, with verification of the knockdown efficiency by western blotting and RT-PCR (Figure 5A). First, it was found that AOAA treatment had no significant effect on the proliferation of PDAC cells in vitro (Figure S3A). We then co-cultured CAFs with PANC-1 or MIAPaCa-2 cells, and found that inhibition of stromal CBS (shCBS or AOAA treatment) resensitized ferroptosis evasion induced by Erastin and IKE in the PDAC cell lines, and this resensitization was negated by cysteine supplementation (Figure 5B-C, Figure S3B-C). In addition, both stromal CBS-knockdown and AOAA treatment significantly inhibited tumor proliferation in both PANC-1 and MIAPaCa-2 cells in Erastin/IKE-containing medium, as shown by EdU staining (Figure 5D-E, Figure S3D-E). This stromal CBS-dependent resistance to ferroptosis was confirmed by colony formation assays (Figure 5F-G, Figure S3F-G).

To assess whether the specific suppression of cysteine synthesis in CAFs can modulate tumor ferroptosis resistance in vivo, we employed a heterotopic xenograft mouse model of PDAC in which human PDAC cells were co-implanted with human CAFs, followed by Erastin treatment. In the animals fed a cysteine-free diet, PANC-1 tumors derived from the co-implantation with control CAFs (shCtrl or vehicle) showed significantly increased tumor growth relative to tumors co-implantation with CBS-depleted CAFs (shCBS or AOAA treatment), while this phenomenon was significantly abolished by supplementing cysteine in the mouse diet (Figure 5H, Figure S4H). We next investigated the proliferative capacity and lipid peroxidation of the tumors using Ki-67 and 4-HNE staining, which confirmed that the cysteine-containing diet reversed the effects of stromal CBS depletion on tumor proliferation and lipid peroxidation (Figure 5I, Figure S3I). Altogether, these data demonstrate that modulation of de novo cysteine synthesis in CAFs leads to a reduction in ferroptosis resistance in PDAC.

### Pancreatic cancer cells require exogenous cysteine-dependent GSH synthesis to avert ferroptosis

**Figure 6:**
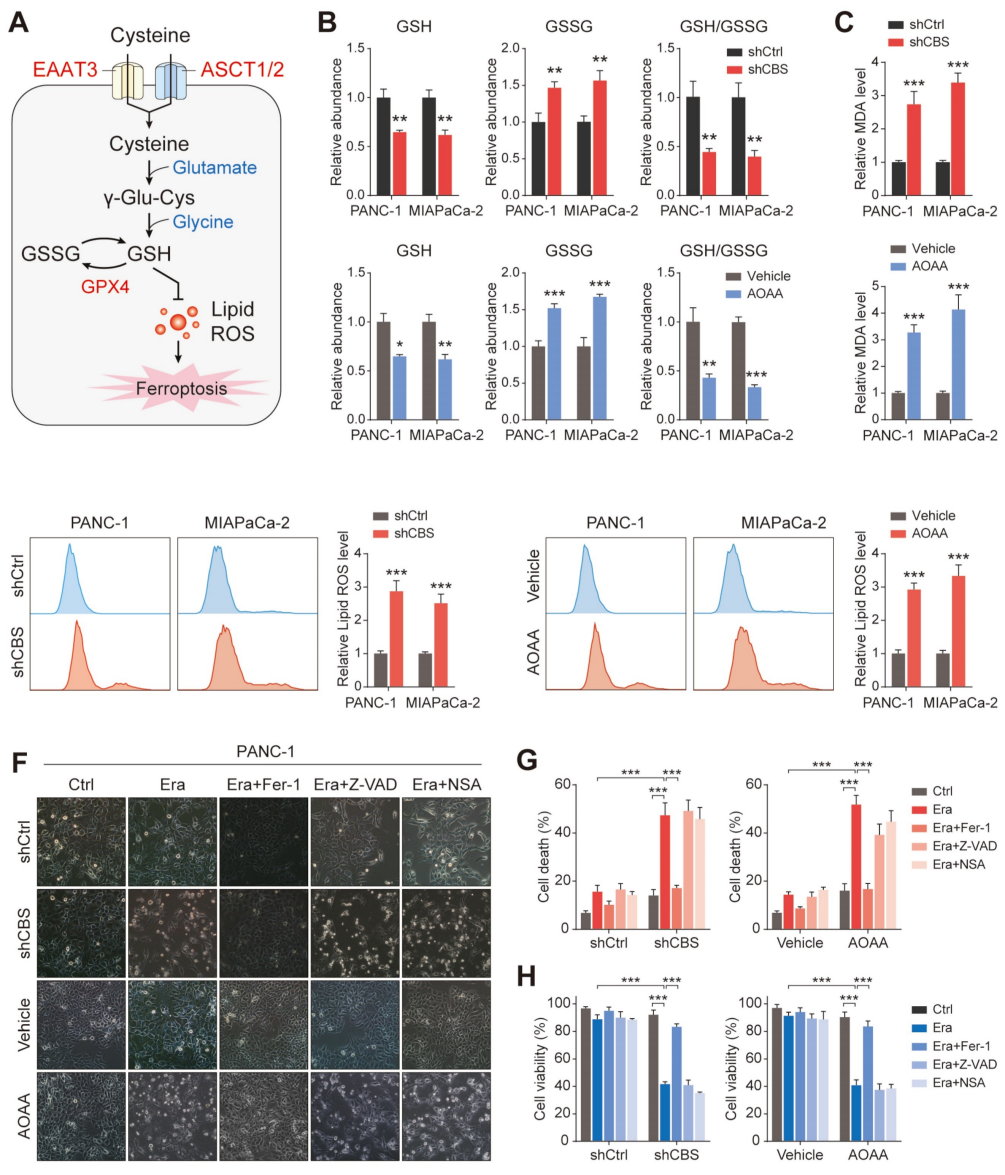
** Pancreatic cancer cells require exogenous cysteine-dependent GSH synthesis to avert ferroptosis.** (A) Cysteine transport and ferroptosis mechanisms. (B) GSH levels. (C) MDA contents. (D) ROS levels, shown by flow cytometry. (E) Micrographs showing cell death after drug treatment. (F) Cell death shown by flow cytometry. (G) Cell viability shown by CCK-8 assays. * P < 0.05, ** P < 0.01, *** P < 0.001.

Cysteine is imported via the excitatory amino acid transporter 3 (EAAT3) and the alanine, serine, cysteine transporter (ASCT)^24^, after which it is used by various metabolic pathways, including the production of sulfur-containing molecules such as glutathione (Figure 6A). Therefore, we mined TCGA transcriptome datasets to investigate the expression of EAAT3 and ASCT1/2, finding that both EAAT3 and ASCT2 showed increased expression in tumor tissues, indicating enhanced cysteine uptake in PDAC (Figure S4A). To identify the main transporter of cysteine in PDAC, we disrupted SLC7A11, EAAT3, ASCT1 and ASCT2 in two PDAC cell lines by siRNAs and the knockdown efficiency of the siRNAs used were validated (Figure S4B). First, targeting the individual transporters did not present any significant effect on cell proliferation (Figure S4C). We then measured the exocrine levels of cysteine in PDAC and CAF co-culture system, and found that only knockdown of ASCT2 significantly interferes with tumor uptake of cysteine (Figure S4D).

We then measured the levels of glutathione species in tumor metabolite fractions to determine whether the tumor-imported cysteine was utilized to maintain GSH levels. The results showed that inhibition of stromal CBS (shCBS or AOAA treatment) significantly reduced both the levels of GSH and the ratio of GSH/GSSG in tumor cells with cystine-free medium (Figure 6B). Given that ferroptosis is characterized by lipid peroxidation, the effects of CAF-secreted cysteine on malondialdehyde (MDA, an end product of lipid peroxidation) and lipid ROS production were investigated using a model in which CAFs were co-cultured with PDAC cells. Inhibition of stromal CBS was found to increase lipid peroxidation in PANC-1 and MIAPaCa-2 cells grown in cysteine-free medium (Figure 6C-D).

Functionally, inhibition of stromal CBS significantly promoted Erastin-induced cell death, which could be prevented by ferrostatin-1 (Fer-1, a ferroptosis inhibitor) in different PDAC cells. In contrast, both a cell death inhibitor (Z-VAD-FMK) and a necroptosis inhibitor (necrosulfonamide, NSA) failed to suppress the Erastin-triggered increase in cell death (Figure 6E-F, Figure S5A, B). Cell viability analysis further confirmed that only Fer-1 abolished stromal CBS-dependent PDAC ferroptosis (Figure 6G, Figure S5C). Furthermore, measurement of lipid ROS, MDA, and GSH levels in Erastin-treated PDAC cells revealed that Fer-1 also rescued lipid peroxidation induced by stromal CBS inhibition (Figure S5D-F). In addition, we investigated whether intracellular GSH synthesis in tumor cells is essential for cysteine-mediated ferroptosis resistance. Using an inhibitor of GCLC, buthionine sulfoximine (BSO), we found that BSO treatment significantly decreased intracellular GSH level, increased Erastin-induced cell death and inhibited proliferation in tumor cells co-cultured with CBS-disrupted CAFs (Figure S6A-C). These findings suggest that CAF-secreted cysteine serves as an important metabolic substrate for glutathione synthesis, which is a key weapon in averting PDAC ferroptosis.

### CAFs release cysteine contributing to cisplatin resistance of PDAC

**Figure 7:**
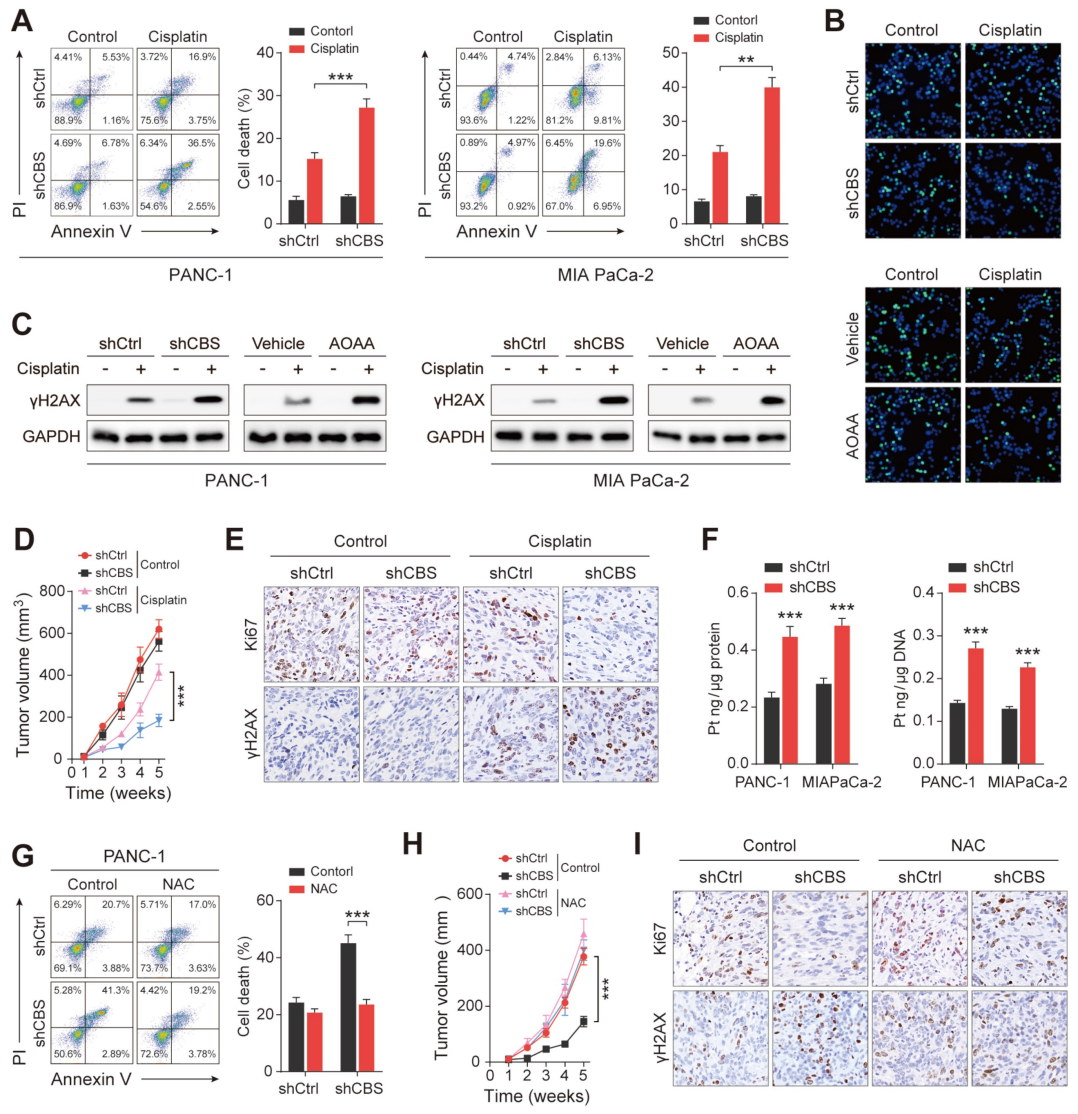
** CAFs release cysteine leading to cisplatin resistance in PDAC.** (A) Cell death, shown by flow cytometry. (B) Proliferation shown by EdU assays. (C) Expression of γH2AX in cells, shown by western blotting. (D) Mouse tumor size statistical chart. (E) Immunohistochemistry of tumor tissues showing expression of Ki-67 and γH2AX. (F) Intracellular cisplatin contents shown by CP-MS. (G) Cell death shown by flow cytometry. (H) Statistical chart of tumor size in mice after NAC treatment. (I) Immunohistochemical detection of Ki-67 and γH2AX expression in mouse tumor tissues after NAC treatment. ** P < 0.01, *** P < 0.001.

Substantial evidence has demonstrated that fibroblasts reduce the accumulation of cisplatin in ovarian cancer cells by releasing GSH and cysteine, resulting in resistance to platinum-based chemotherapy^25^. Moreover, previous results showed that the presence of stromal fibroblasts contributed significantly to platinum resistance in PDAC cells (Figure 2B). We next investigated the role of CAF-released cysteine in cisplatin resistance in PDAC. After excluding the effect of platinum on the proliferative activity of CAFs (Figure S7A), PDAC cells were co-cultured with CAFs in vitro, finding that CAF-released cysteine protected tumor cells from cisplatin-induced cell death as shown by the reduced proportion of Annexin V^+^ tumor cells (Figure 7A). EdU staining assays confirmed the reduced proliferation of PDAC cells in the presence of CBS-depleted CAFs (Figure 7B). Cisplatin leads to DNA crosslinking and stimulates γH2AX phosphorylation to generate γH2AX, which is a major marker for DNA damage^26^. Therefore, we examined the expression of cisplatin-triggered γH2AX in co-cultured tumor and stromal cells. The results showed that depletion of CBS in stromal cells reduced the levels of cisplatin-triggered γH2AX in PDAC cells (Figure 7C). Next, a mix of PANC-1 cells and CAFs was injected into BALB/c-Nude mice, followed by treatment of the mice with cisplatin. This showed that the tumor volumes were increased in the cisplatin-treated mice injected with PANC-1 cells and CBS-knockdown CAFs, compared with cisplatin-treated mice injected with CBS-control CAFs (Figure 7D). Immunofluorescence staining confirmed that CBS-knockdown CAFs also reduced the levels of Ki67 and increased those of γH2AX in cisplatin-treated tumors (Figure 7E).

Intracellular GSH levels have been suggested to regulate platinum sensitivity by decreasing DNA platinum accumulation in cultured tumor cells^25^. To elucidate the molecular mechanism underlying fibroblast-mediated cisplatin resistance, inductively coupled plasma-high resolution mass spectrometry (ICP-MS) analysis was performed, showing that the intracellular cisplatin content was reduced in PDAC cells in the presence of CBS-knockdown CAFs (Figure 7F). Reduced cisplatin contents were also observed in the genomic DNA of cisplatin-treated PDAC cells (Figure 7F). Next, N-acetyl cysteine (NAC), a GSH precursor, was used to investigate the effect of intracellular GSH levels on cisplatin-induced tumor cell death. Elevated GSH levels were observed in co-cultured PDAC cells after NAC treatment (Figure S7B) while NAC treatment counteracted the increased cell death and decreased proliferation seen in tumor cells co-cultured with CBS-disrupted CAFs (Figure 7G, Figure S7C-D). As expected, we also found that NAC treatment reduced the expression of cisplatin-induced γH2AX and decreased the platinum content of genomic DNA in the tumor cell co-culture model (Figure S7E-F). Moreover, we observed that NAC treatment significantly eliminated the difference in tumor growth seen between the shCtrl and shCBS groups (Figure 7H). Immunofluorescence staining also showed no significant differences in the Ki67 and γH2AX levels between the two groups (Figure 7I). We further confirmed that CAF-dependent GSH synthesis reduced the accumulation of the chemotherapy drug in tumor cells from cisplatin-treated mice (Figure S7G). Thus, CAFs confer cisplatin-resistance in PDAC cells by reducing the accumulation of intracellular cisplatin.

### TGF-β/SMAD5 pathway activates expression of CBS/CTH in CAFs

To identify the mechanism that regulates transsulfurantion pathway activity in CAFs, we first attempted to transform PSCs into CAFs by culturing them in PDAC-conditioned medium (CM) and found that PDAC-CM significantly increased expression of CBS, CTH and activated CAF marker (Figure S8A-C). Moreover, PDAC-CM treatment also enhanced the expression of CBS in CAFs (Figure S8D). These results indicated that upstream signals that governed activity of transsulfurantion metabolism may originate from PDAC cells.

**Figure 8:**
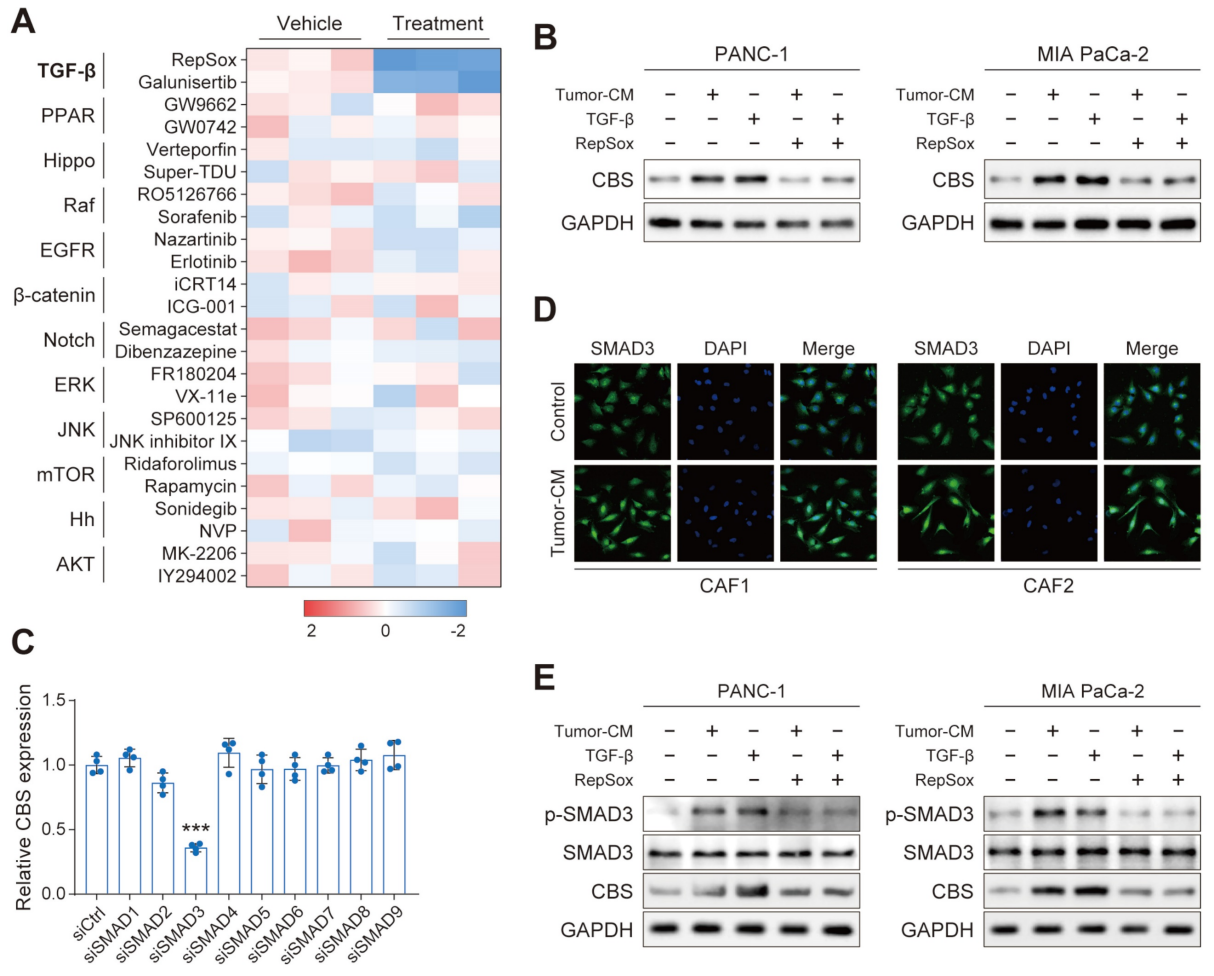
** TGF-β/SMAD5 pathway activates expression of CBS/CTH in CAFs.** (A) Cells were treated with various signaling factors followed by measurement of cellular CBS mRNA expression levels using RT-PCR. (B) Expression of CBS after TGF-β and RepSox antagonist treatment shown by western blotting. (C) Expression of CBS in cells after siRNA treatment, shown by RT-PCR. (D) SMAD3 expression shown by immunofluorescence. (E) Effects of PDAC-CM on TGF-β/SMAD3 and CBS shown by western blotting. *** P < 0.001.

We next performed a screening in PANC-1 using a panel of compounds targeting various signals, including the TGF-β, YAP, and EGFR signals, among others. Interestingly, the CBS mRNA level of PDAC was dramatically reduced only by treatment with TGF-β inhibitor of RepSox and galunisertib (Figure 8A). We further verified that CBS was stimulated in PDAC cells treated with PDAC-CM or TGF-β, and such effect was antagonized by RepSox (Figure 8B). Previous studies have provided evidence that Smad signaling uniquely defined canonical signaling in response to TGF-β activation^8^. Therefore, we screened SMAD proteins (SMAD1-SMAD9) in vitro using individual small interfering RNAs (siRNAs) and found that SMAD3 knockdown maximally reduced the expression of CBS in CAFs (Figure 8C). Immunofluorescence and western blot assay further confirmed that PDAC-CM derived TGF-β/SMAD3 signaling up-regulated stromal CBS activity (Figure 8D-E).

### Stromal transsulfuration pathway is regulated by TGF-β/SMAD3/ATF4 signaling

**Figure 9:**
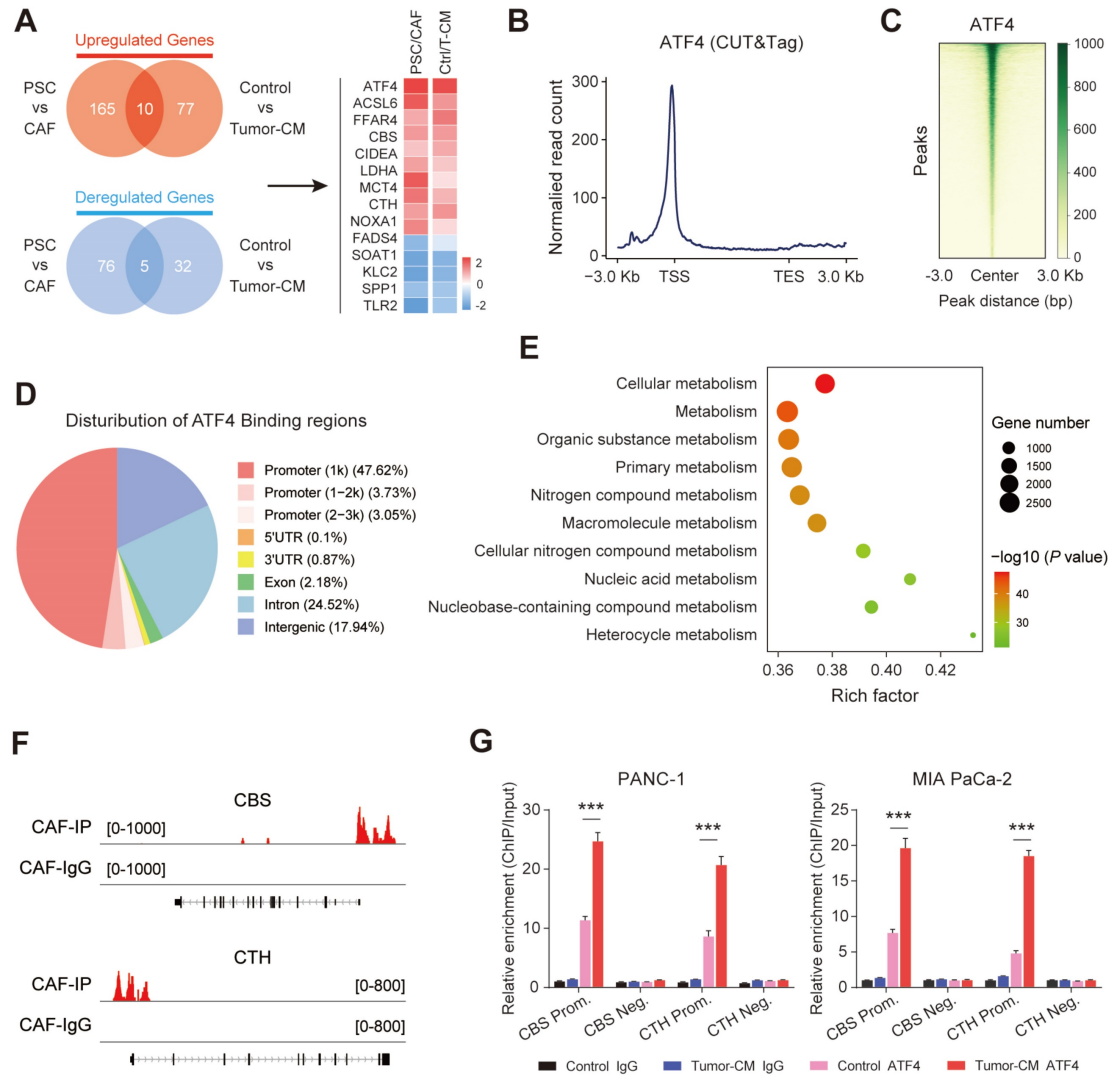
**Stromal transsulfuration pathway is regulated by TGF-β/SMAD3/ATF4 signaling.** (A) Transcriptionomic up-regulation gene intersection analysis of the two groups. (B) Reads were mainly enriched near the TSS. (C) Heatmap of the central peak signals indicating that the signals of the enriched sites were concentrated in the TSS region. (D) Pie chart showing the distribution of peaks in the functional regions of the genes. (E) Gene Ontology (GO) analysis results showing the pathways in which target genes may be enriched. (F) IGV software visualization showing the CBS and CTH-enriched peaks. (G) ChIP enrichment analysis of ATF4.

Transcriptomic analysis was further performed on two groups of fibroblasts (PSCs/CAFs and CAF-control/CAF-Tumor-CM). The results showed that a total of 15 overlapping genes were differentially expressed in the two groups, and AFT4 ranked the top among these up-regulated genes (Figure 9A). Therefore, we hypothesized that PDAC-secreted TGF-β activated CAF transsulfuration pathway via SMAD3/ATF4 signaling. To verify this hypothesis, we first confirmed that RepSox and SIS3 (a specific inhibitor of SMAD3) antagonized the up-regulation of ATF4, CBS and CTH expression in PDAC-CM treated CAFs (Figure S9A). In addition, the protein and mRNA levels of ATF4 were significantly increased in CAFs compared with matched PSCs (Figure S9B).

ATF4, a stress-induced transcription factor, controls the expression of a wide range of adaptive genes that allow cells to endure periods of stress^27^. we speculated that ATF4 may be able to bind promoter regions of CBS/CTH and transcriptionally activate their expression in CAFs. CUT&Tag was performed in CAFs to identify downstream targets of ATF4. We found that considerable strong ATF4 signals across the entire set of sites, particularly at TSSs (Figure 9B). In addition, the signal values of all peaks were meticulously analyzed, and a CUT&Tag heatmap was generated to demonstrate that the signals were strongly concentrated near enriched sites (Figure 9C), with mainly ATF4 signal distributed across gene promoter region (promoter ≤1 Kb) (Figure 9D). We applied gene set enrichment analysis (GSEA) to the CUT&Tag dataset, and the Gene Ontology (GO) analysis indicated that the ATF4 signature was mainly enriched in cellular metabolic pathway (Figure 9E). Furthermore, CUT&Tag sequencing data indicated robust enrichment of ATF4 in the promoters of CBS and CTH (Figure 9F). The ChIP-qPCR results further demonstrated that ATF4 was specifically occupied the promoter regions of CBS and CTH, and that the signature of ATF4 binding was further enhanced when cells were treated with PDAC-CM (Figure 9G).

We next examined the role of TGF-β/SMAD3/ATF4 signaling in promoting ferroptosis and cisplatin resistance in PDAC. CAF-secreted cysteine measurement showed that PDAC-CM enhanced cysteine secretion, and this effect was abrogated by inhibition of TGF-β/SMAD3/ATF4 signaling with RepSox, SIS3 or siATF4 treatment (Figure S10A). We further cultured PDAC cells in CAF medium with different treatment conditions, and found that activation TGF-β signaling reinforced ferroptosis and cisplatin resistance in PDAC cells, which phenotype was reversed by RepSox, SIS3 or siATF4 treatment (Figure S10B-D). Collectively, these results indicate that the TGF-β/SMAD3/ATF4 signaling is involved in regulating the stromal transsulfuration pathway in CAFs.

## Discussion

The tumor microenvironment (TME) is a multicellular system characterized by complex tumor-stromal interactions^3^. CAFs are the most abundant cells within the TME and are strongly associated with cancer progression. CAFs regulate the biology of tumor and other stromal cells through direct cell-to-cell contact, resulting in the release of many regulatory factors and synthesis and remodeling of the ECM, thus influencing both cancer development and treatment resistance^6^. Recent reports suggest that stromal cells play an important role in alleviating nutrient deprivation in PDAC cells. For example, CAFs are critical for PDAC metabolism due to their secretion of alanine, which replaces glucose and glutamine-derived carbon in PDAC, thereby driving the tricarboxylic acid (TCA) cycle and promoting the biosynthesis of non-essential amino acids and lipids^28^. Others have suggested that CAFs significantly increase glucose utilization and promote PDAC growth during glutamine deprivation resulting from nucleoside secretion during autophagy in a nuclear-fragile X retardation interaction protein 1 (NUFIP1)-dependent manner^29^. In addition, glutathione and cysteine released from fibroblasts have been reported to reduce the nuclear accumulation of platinum in ovarian cancer cells, leading to resistance to platinum-based chemotherapy^25^. In this study, using a pancreatic cancer model system and in vivo co-transplantation assays, it was found that CAFs have significant metabolic crosstalk with pancreatic cancer cells and provide a metabolic basis for the development of pancreatic cancer. A specific role for CAF-derived cysteine was also observed, which plays a vital role in the redox homeostasis of PDAC and is a component of the major antioxidant GSH.

Ferroptosis is a form of cell death induced by a massive buildup of lipid ROS^30^. Tumor cells are prone to ferroptosis due to their unique metabolism, high ROS loads, and specific mutations, and thus contribute to tumorigenesis, tumor progression, metastasis, and treatment resistance^18^. At the same time, tumorigenesis and tumor progression are accompanied by increasing levels of ROS and altered expression of antioxidants^31^. This carcinogenic signaling increases lipid ROS generation in numerous tumor types and is inhibited by cysteine-derived metabolites. The system xC^-^ antiporter (SLC7A11) converts external oxidized cysteine (cystine) to intracellular glutamate to obtain the majority of cellular cysteine. Cysteine is stable in the acidic and hypoxic environment of the tumor, implying that added EAAT3/ASCT1/ASCT2-recessive cysteine is also important^32^,. In this study, we found that the presence of CAFs protected against PDAC after treatment with the ferroptosis inducers Erastin and RSL3; however, Erastin resistance to CAF-induced PDAC was stronger than that of RSL3. This indicates that changes in the intermediate metabolic pathways associated with ferroptosis may play a significant role in this outcome^33^. Further research revealed that cysteine, which is largely generated by CAFs, contributed to CAF-induced ferroptosis resistance in PDAC cells, indicating that inhibition of SLC7A11 has little effect on cysteine absorption in tumors.

Recent studies have shown that most cancer cell lines have intact transsulfurization pathways that respond to changes in cysteine levels in the environment^34^. When the external source of cysteine is limited, cancer cells activate the cysteine biosynthesis pathway via reverse sulfur transthiosification, promoting tumor survival and growth^34^. In contrast, we discovered that the PDAC-closed transsulfurization pathway was downregulated in both CAFs and normal pancreatic cells. We investigated how PDAC avoids oxidative damage in the presence of cystine deficiency and discovered a mutually beneficial link between cysteine metabolism in CAFs and PDAC cells. Thus, the findings show that cysteine released by CAFs plays a critical role in balancing oxidative damage and antioxidant defense in the cystine-hungry pancreatic milieu.

Drug resistance to cancer treatment is common and often leads to tumor growth. Platinum-based chemotherapy has recently received increased attention due to the discovery that germline or somatic pathogenic mutations in PDAC, BRCA, or PALB2 are highly responsive to platinum-based chemotherapy and poly(adenosine ribose) polymerase (PARPi) therapy^35^, ^36^. The platinum molecule forms crosslinks with the purine base of DNA and disrupts replication and transcription, causing DNA damage and degradation^25^, ^37^. Many studies have been conducted to investigate the responsiveness of stromal CAFs to anticancer treatments as well as their involvement in conferring therapeutic resistance. Recently, drug clearance by fibroblasts has emerged as a unique mechanism underlying nuclear cisplatin accumulation in ovarian cancer, which lowers the amount of active cisplatin due to intratumor GSH, limiting the quantity of drug available to tumor cells^25^. CAFs can also increase glutathione levels and reduce ROS production to prevent chemotherapy-induced prostate cancer cell death^38^. We discovered that fibroblasts release cysteine, increase intracellular GSH levels, and reduce the accumulation of platinum in DNA in PDAC cells. Thus, our findings provide an unexpected metabolic paradigm in drug resistance, by which fibroblasts affect sulfhydryl metabolism and thus influence the sensitivity of tumor cells to anticancer treatment.

Cancer cells have been shown to transform stromal fibroblasts into cells with a protumor phenotype. This is dependent on a variety of tumor-derived substances, as well as specific TME stimuli, such as local hypoxia and oxidative stress^39^. TGF-β, PDGF, and IL-6 are known fibroblast-activating factors that participate in cell signaling pathways via their associated receptors^40^, ^41^. We screened a variety of chemicals targeting different signals in PDAC cells to identify upstream signals that control CBS/CTH expression and hence potentially alter transthiocysteine production pathways. Interestingly, we found that CBS mRNA levels were significantly reduced when TGF-β signaling was inhibited by RepSox or galunisertib. TGF-β has been studied and found to be widely produced by almost all cell types, including PDAC, and the TGF-β signaling pathway has been shown to have pleiotropic effects on CAF behavior through its receptor and Smad proteins.

ATF4 is one of the key regulators of the cellular stress response, facilitating cellular adaptation to a limited nutrient environment, and available evidence suggests that ATF4 signaling is critical for the maintenance of metabolic homeostasis in tumor cells via the sulfur pathway^34^, ^42^, making it a novel target for anti-tumor strategies. In our study, transcriptomic and ChIP analyses were performed, showing that the ATF4 transcription factor acts as an upstream link in the activation of CAF CBS/CTH expression. Thus, we demonstrate that TGF-β/SMAD3/ATF4 signaling is an integral upstream pathway involved in the regulation of the matrix sulfur transport pathway in CAFs.

## Conclusions

In summary, the findings of this study demonstrated that TGF-β/SMAD3/ATF4 signaling mediated the transsulfuration pathway in CAFs, leading to drug resistance in PDAC. Targeting the matrix sulfur transport pathway may thus be worth investigating as a therapeutic strategy to overcome desferrioxicosis.

## Supplementary Material

Supplementary figures and table.
