# Supplementary material for: Cancer-associated fibroblasts reprogram cysteine metabolism to increase tumor resistance to ferroptosis in pancreatic cancer
Source: Theranostics. 2024 Feb 11;14(4):1683–700. doi: 10.7150/thno.89805 (PMC10879865; doi:10.7150/thno.89805)
Supplement: Supplementary file 1 — Supplementary figures and table. [file thnov14p1683s1.pdf]

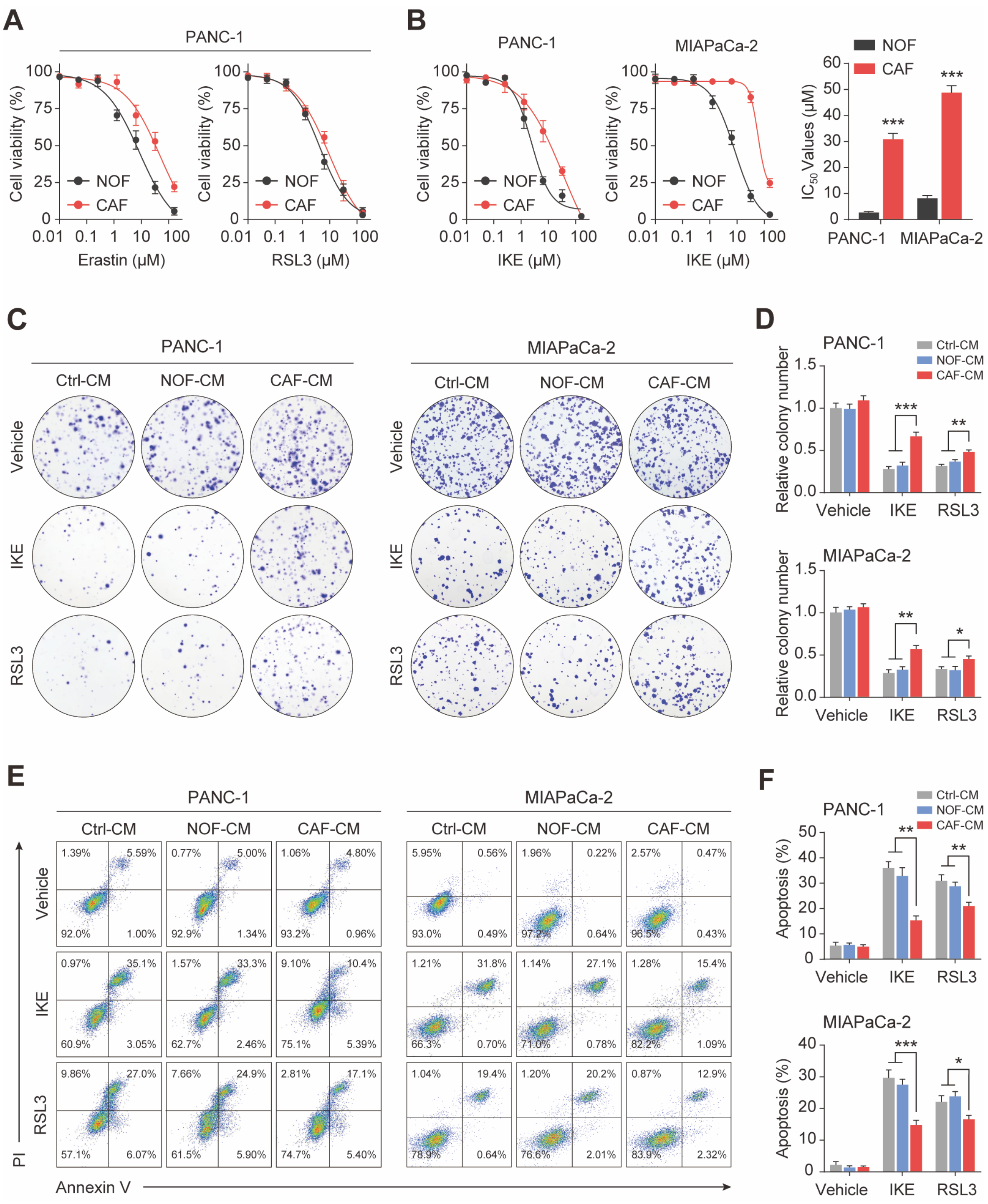

Figure S1. CAFs are associated with ferroptosis resistance in PDAC. PDAC cells were cultured with CAF-CM or PCS-CM, and after (A-B) Erastin, RSL3, or IKE treatment, CCK-8 was used to measure cell viability. (C-D) Colony formation by PANC-1 and MIAPaCa-2 cells was evaluated by clonal formation assay after treatment with vehicle, RSL3, or IKE. (E-F) Cell death ratios in PANC-1 and MIAPaCa-2 cells were evaluated by cell death assays after treatment with vehicle, RSL3, or IKE. * $P < 0.05$, ** $P < 0.01$, *** $P < 0.001$.

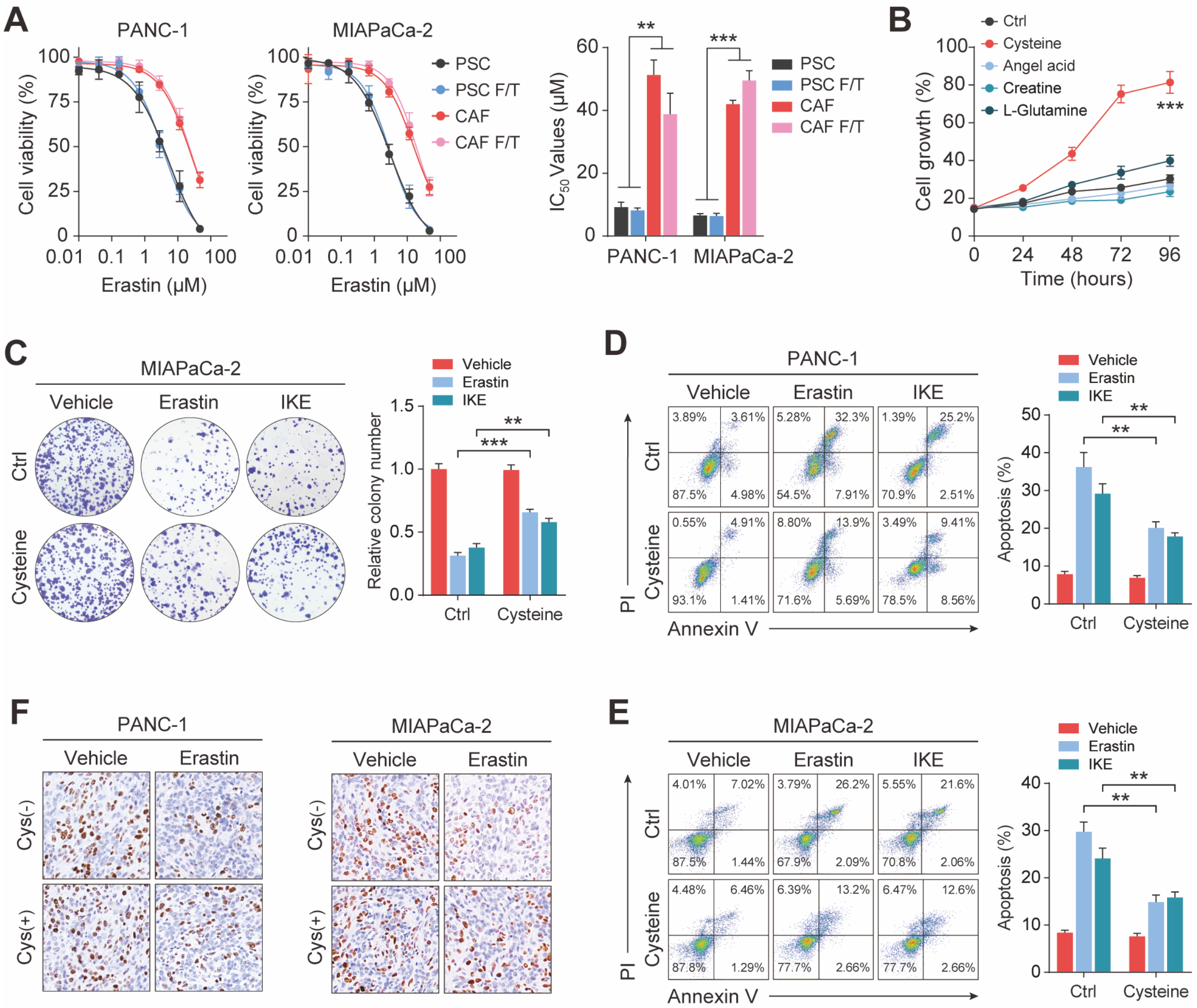

Figure S2. Exocrine cysteine was essential for CAF-induced ferroptosis resistance in PDAC. (A) Cell viability was measured after treatment with CAF-CM, three repeated freeze-thaw cycles of CAF-CM, PSC-CM, or three repeated freeze-thaw cycles of PSC-CM using Erastin. (B) Cell viability after culture of PANC-1 cells with cysteine, angelic acid, creatine, or glutamine, measured by CCK-8 assays. The MIAPaCa-2 cell line was cultured with Ctrl or cysteine and treated with Erastin, (C) clonal formation assay to assess cell colony formation capacity, and (D-E) Cell death assay to evaluate the cell death ratio of PANC-1 and MIAPaCa-2 cells. (F) Representative IHC staining of Ki67 in mouse xenograft tumors. Scale bar = 100 microns. * P ＜0.05, ** P ＜0.01, *** P ＜0.001.

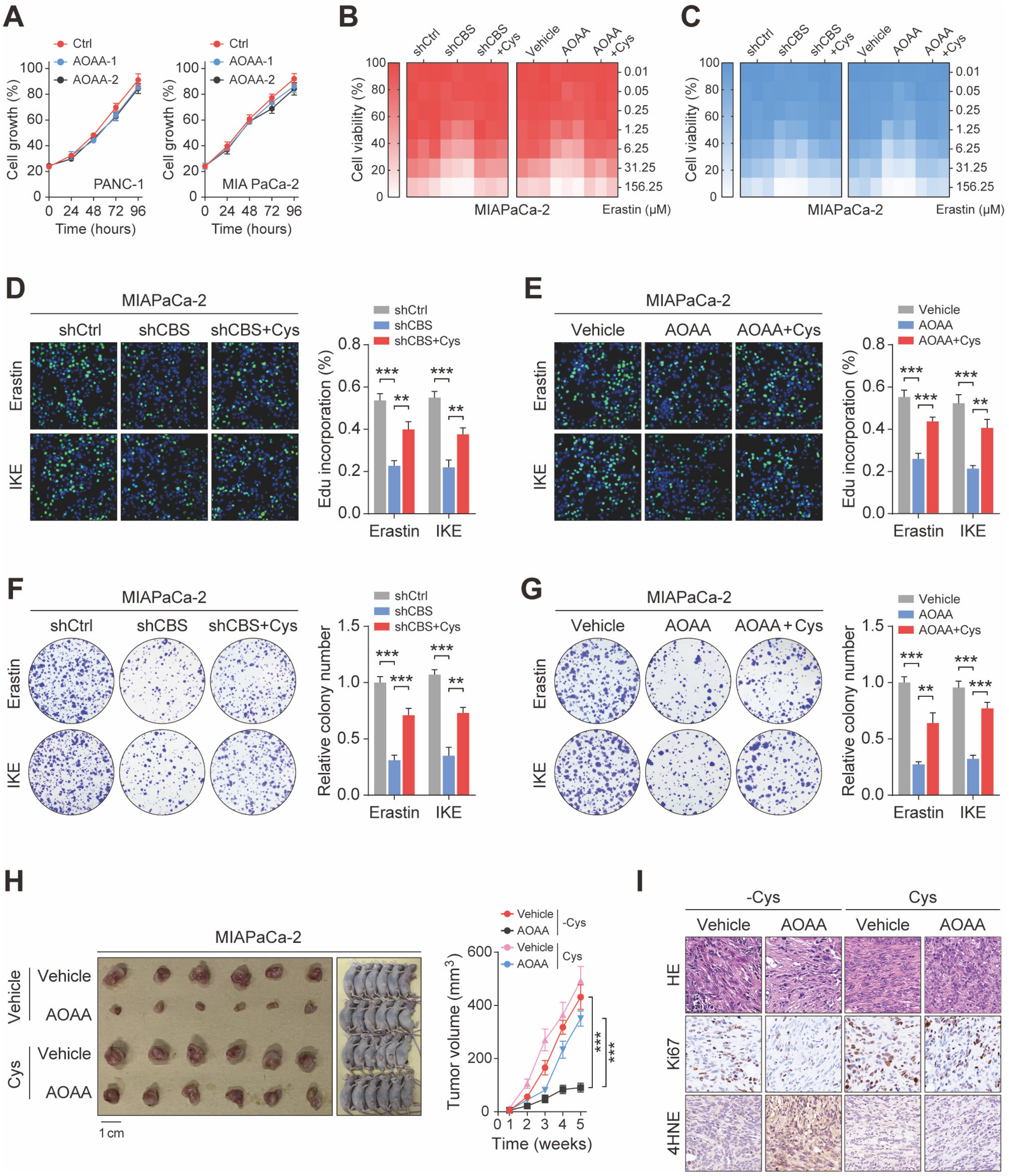

Figure S3. Stromal CBS-dependent de novo biosynthesis of cysteine is required for ferroptosis resistance in PDAC. (A) After treatment of PANC-1 and MIAPaCa-2 with Ctrl, AOAA-1, or AOAA-2, CCK-8 assays were used to measure cell viability. MIAPaCa-2 cells were treated with shRNA or AOAA using Erastin or IKE, (B-C) CCK-8 assays were used to measure cell viability. (D-E) EdU assays were used to measure proliferation. (F-G) colony formation assays. (H) Images and tumor volumes of xenograft tumors (N=6). (I) Representative H&E staining of mouse xenograft tumors and IHC staining of Ki67 and 4HNE. ** P ＜ 0.01，*** P ＜ 0.001.

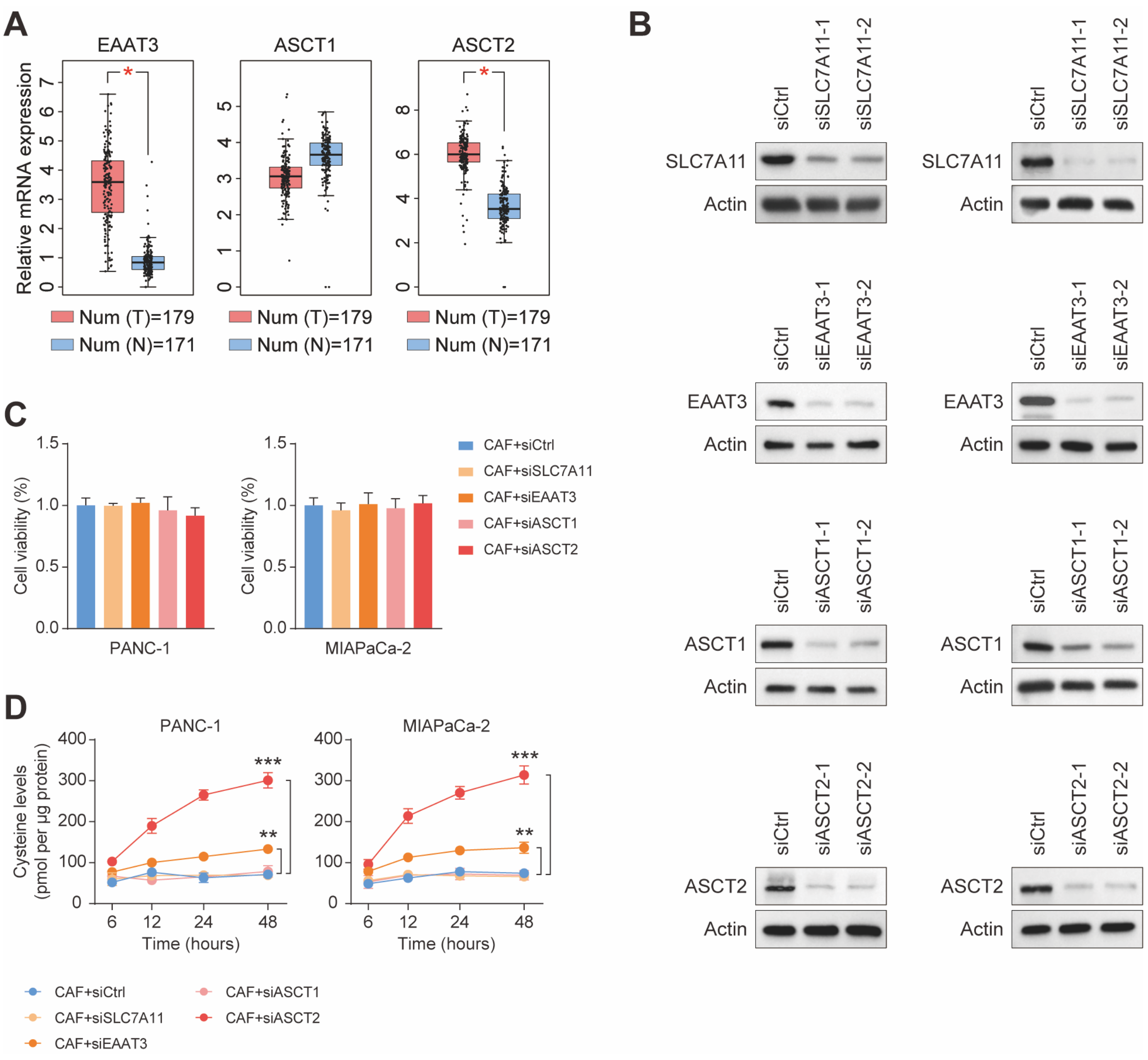

Figure S4. The knockout of ASCT2 significantly impairs the uptake of cysteine by tumors. (A) TCGA database mining of EAAT3, ASCT1, ASCT2 expression in tumor and paracancerous tissues. (B) Expression of SLC7A11, EAAT3, ASCT1 and ASCT2 knockdown efficiency of the siRNAs in cells shown by western blotting. (C) Cell viability in PANC-1 and MIAPaCa-2 cells was measured by CCK-8 assays with siSLC7A11, siEAAT3, siASCT1 or siASCT2. (D) Cysteine levels in PANC-1 and MIAPaCa-2 cells with siSLC7A11, siEAAT3, siASCT1 or siASCT2. * P < 0.05, *** P < 0.001.

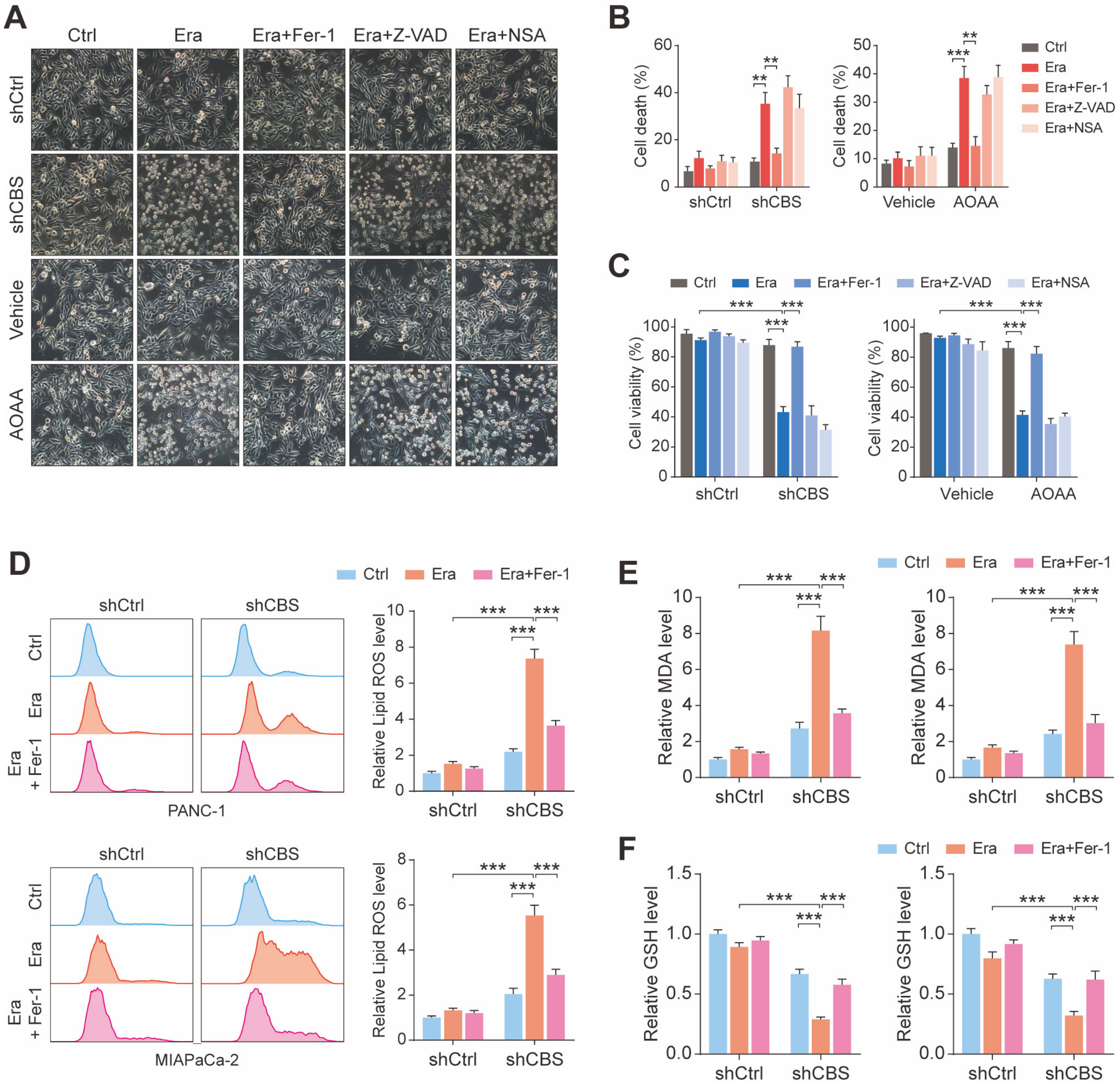

Figure S5. Pancreatic cancer cells require exogenous cysteine-dependent GSH synthesis to avert ferroptosis. (A-B) Micrographs of cell death after drug treatment. (C) Flow cytometry and CCK-8 assays were used to measure cell death and cell viability, respectively. (D) ROS levels, assessed by flow cytometry. (E) MDA contents. (F) GSH levels. T, tumor tissue; N, paracancerous tissue. * $P < 0.05$, ** $P < 0.01$, *** $P < 0.001$.

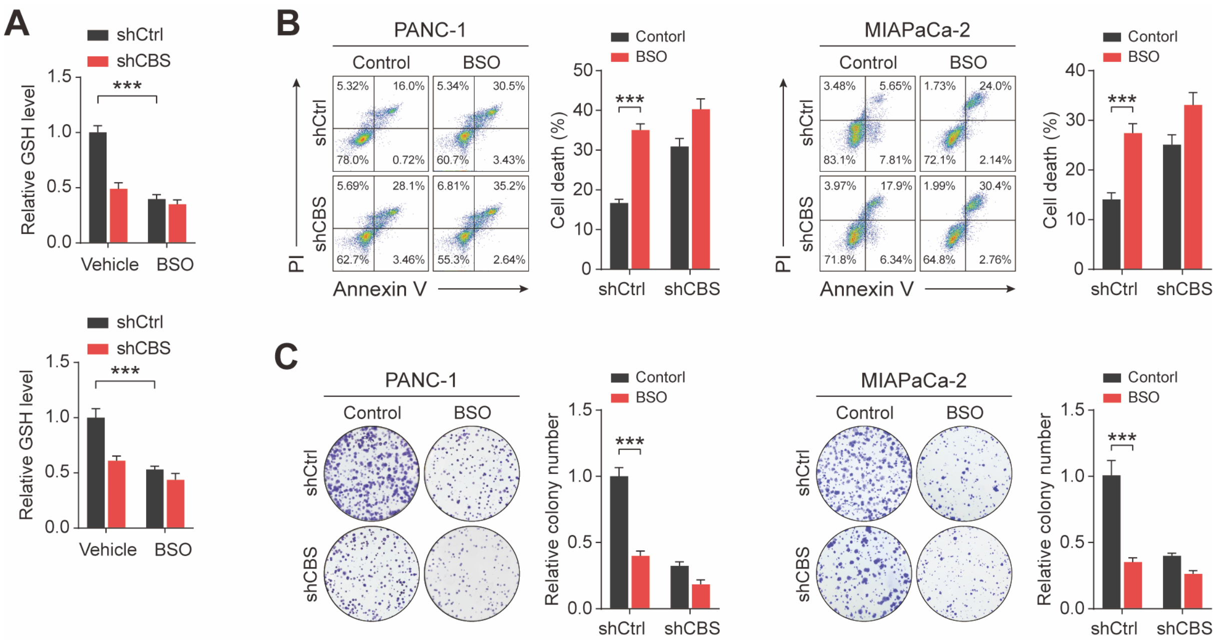

Figure S6. GCLC inhibitor (BSO) decreased intracellular GSH level. (A) GSH levels. (B) Cell death measured by flow cytometry with BSO treatment. (C) colony formation assays with BSO treatment. *** P < 0.001.

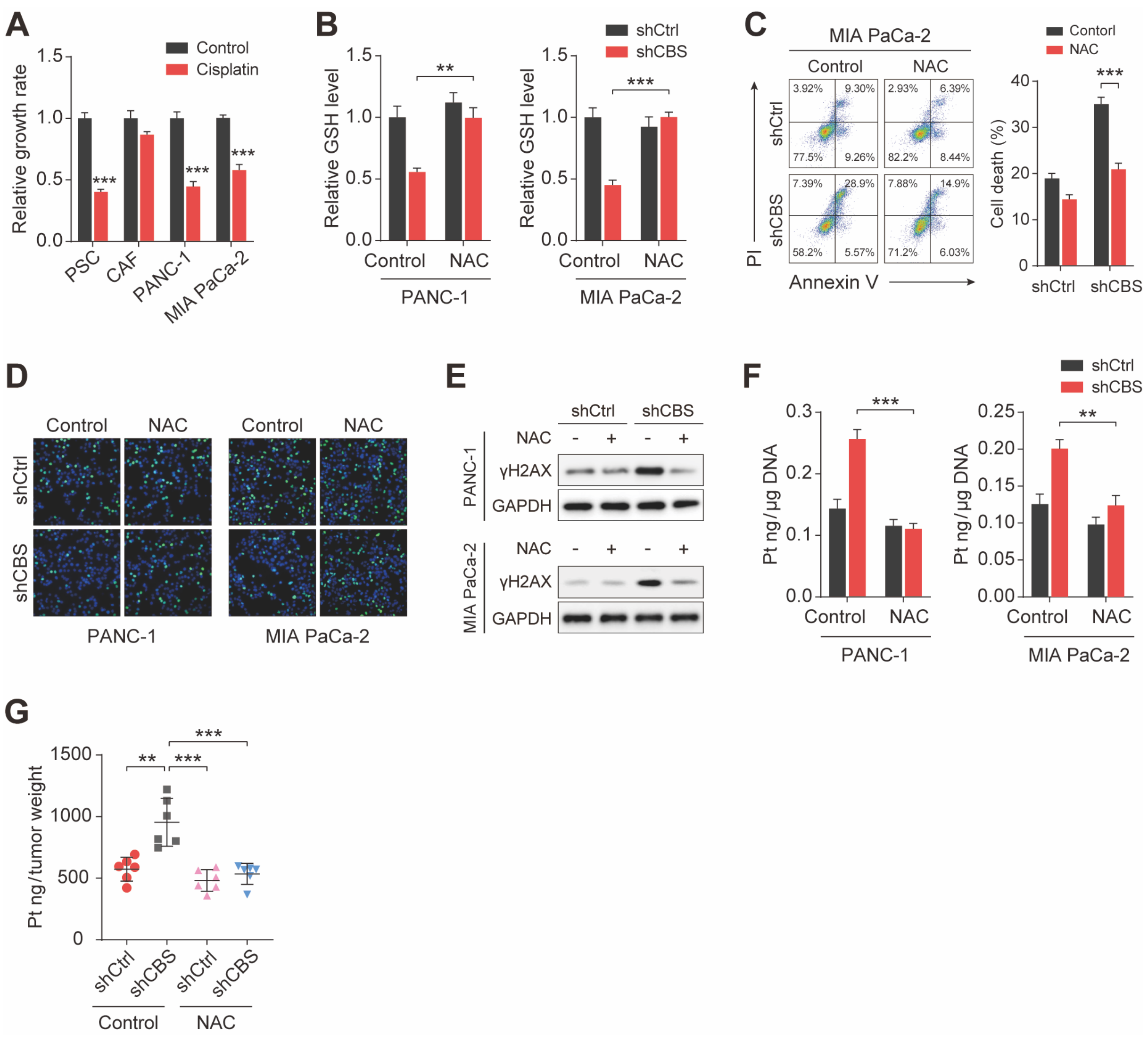

Figure S7. CAFs release cysteine inducing cisplatin resistance in PDAC. (A) Cell viability measured by CCK-8 assays. (B) GSH levels. (C) Cell death measured by flow cytometry. (D) Proliferation measured by EdU assays. (E) Expression of γH2AX in cells shown by western blotting. (F) ICP-MS measurement of intracellular cisplatin content. (G) Statistics on the ratio of cisplatin content to tumor size in tumor tissue. ** P ＜0.01, *** P ＜0.001。

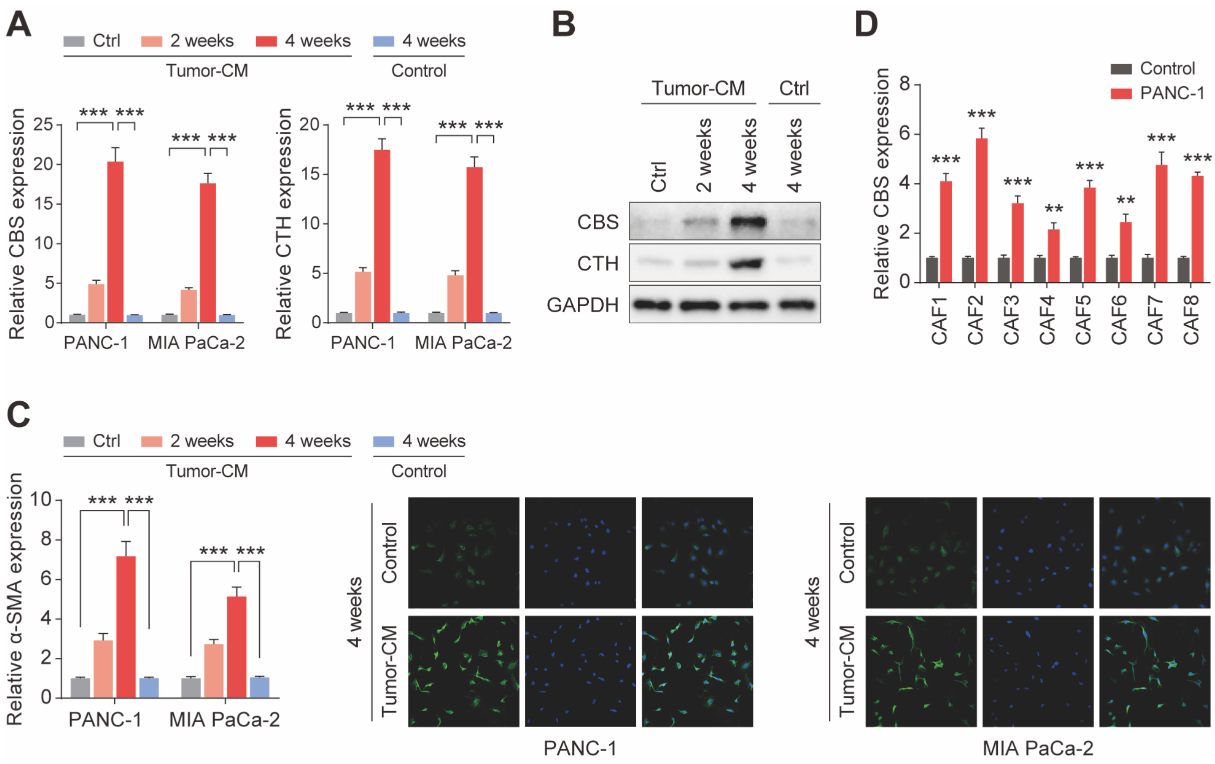

Figure S8. Stromal transsulfuration pathway is regulated by TGF-β/SMAD3/ATF4 signaling. (A) Expression of CBS and CTH in cells shown by RT-PCR. (B) Expression of CBS and CTH in cells shown by western blotting. (C) Expression of α-SMA shown by RT-PCR and immunofluorescence. (D) Expression of CBS in cells after PDAC-CM treatment shown by RT-PCR. ** P < 0.01, *** P < 0.001.

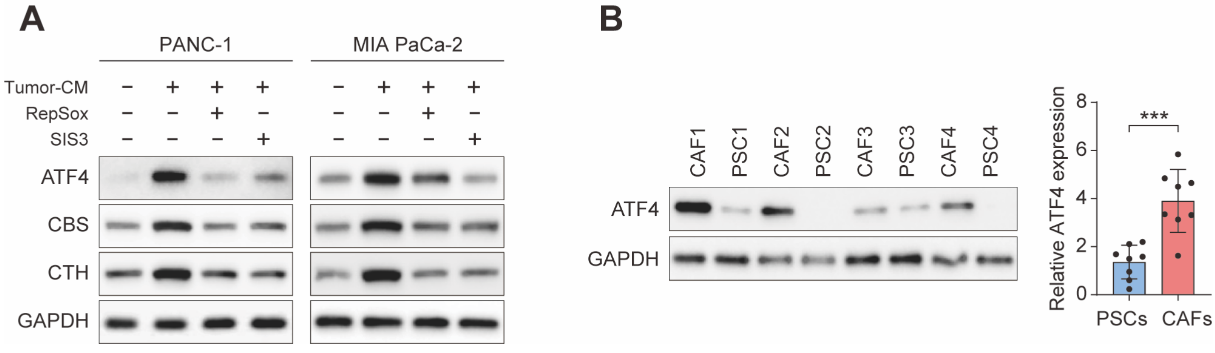

Figure S9. ATF4 were significantly increased in CAFs. (A) Expression of ATF4, CBS, and CTH after TGF-β and RepSox antagonist treatment shown by western blotting. (B) Expression of ATF4 in cells shown by western blotting and RT-PCR. *** P < 0.001.

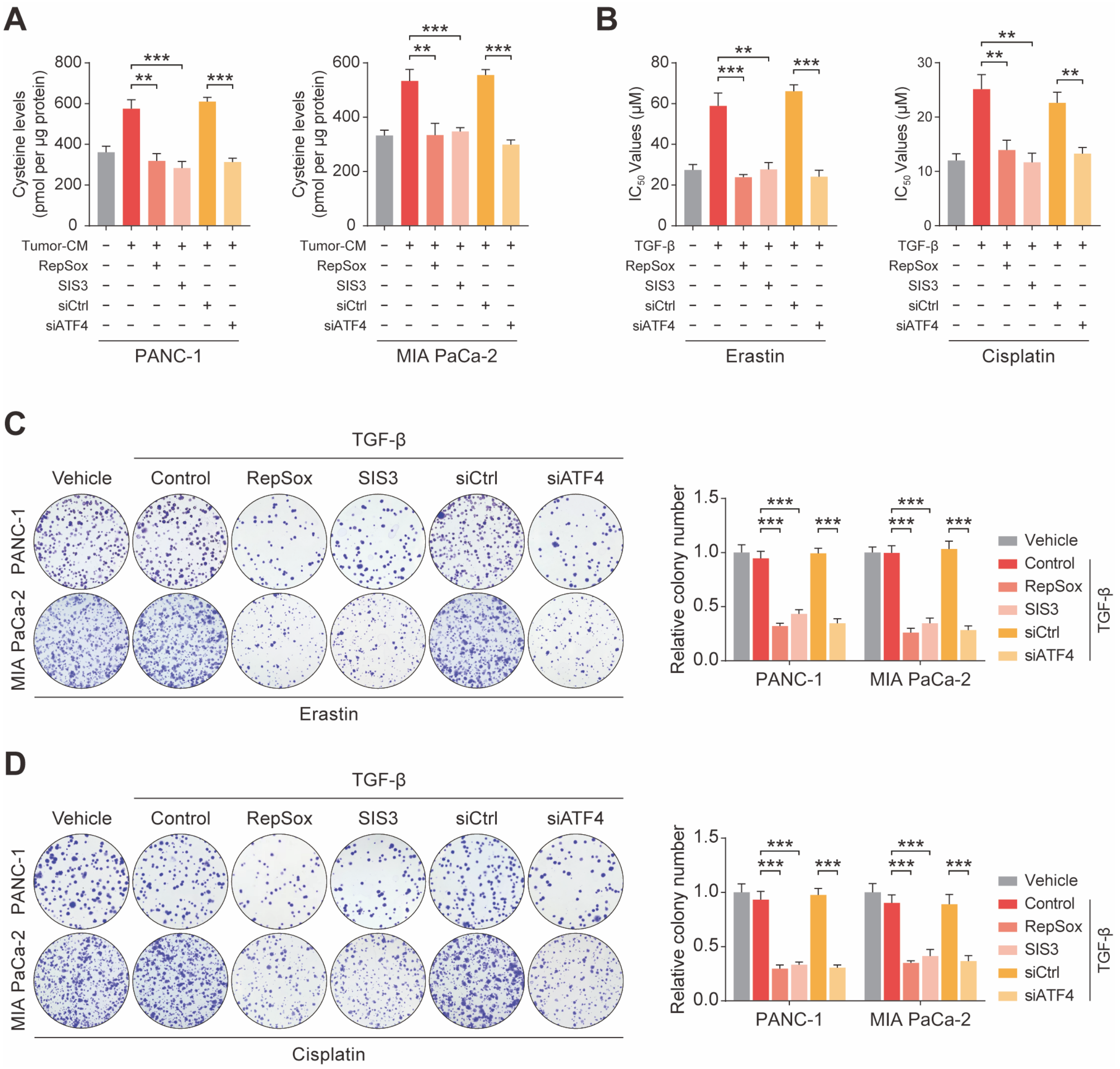

Figure S10. TGF-β/SMAD3/ATF4 signaling pathway promotes ferroptosis and cisplatin resistance in PDAC. (A) Cysteine secretion measured after inhibition of the TGF-β/SMAD3/ATF4 signaling pathway. (B) Cell viability after treatment with different drugs, measured by CCK-8 assays. (C-D) Colony formation by cells after treatment with Erastin or Cisplatin. ** P < 0.01, *** P < 0.001.

Table S1 Primer sequences.

Name	**Forward primer (5' to 3')**	**Reverse primer (5' to 3')**
CBS	ACAGAGCTCACACTTCAGGC	GGTCTGGAGACCGCTGTAAG
CTH	GCATGGGGGTACTTGACACA	TGCACTTTGACTGAGCTCCC
SLC7A11	AAAGCCTGTTGTGTCCACCA	AGAAAATCTGGATCCGGGCG
α-SMA	AGCGTGGCTATTCCTCCGTT	GCAGTGGCCATCTCATTTTCA
ATF4	TCGTCCTGGTGGGATCTAGG	TCTGGCATGGTTTCCAGGTC
β-actin	AGCGAGCATCCCCCAAAGTT	GGGCACGAAGGCTCATCATT
